# Supplementary material for: Centriole distal-end proteins CP110 and Cep97 influence centriole cartwheel growth at the proximal end
Source: J Cell Sci. 2022 Jul 20;135(14):jcs260015. doi: 10.1242/jcs.260015 (PMC9450887; doi:10.1242/jcs.260015)
Supplement: Supplementary information [file joces-135-260015-s1.pdf]

Figure S1

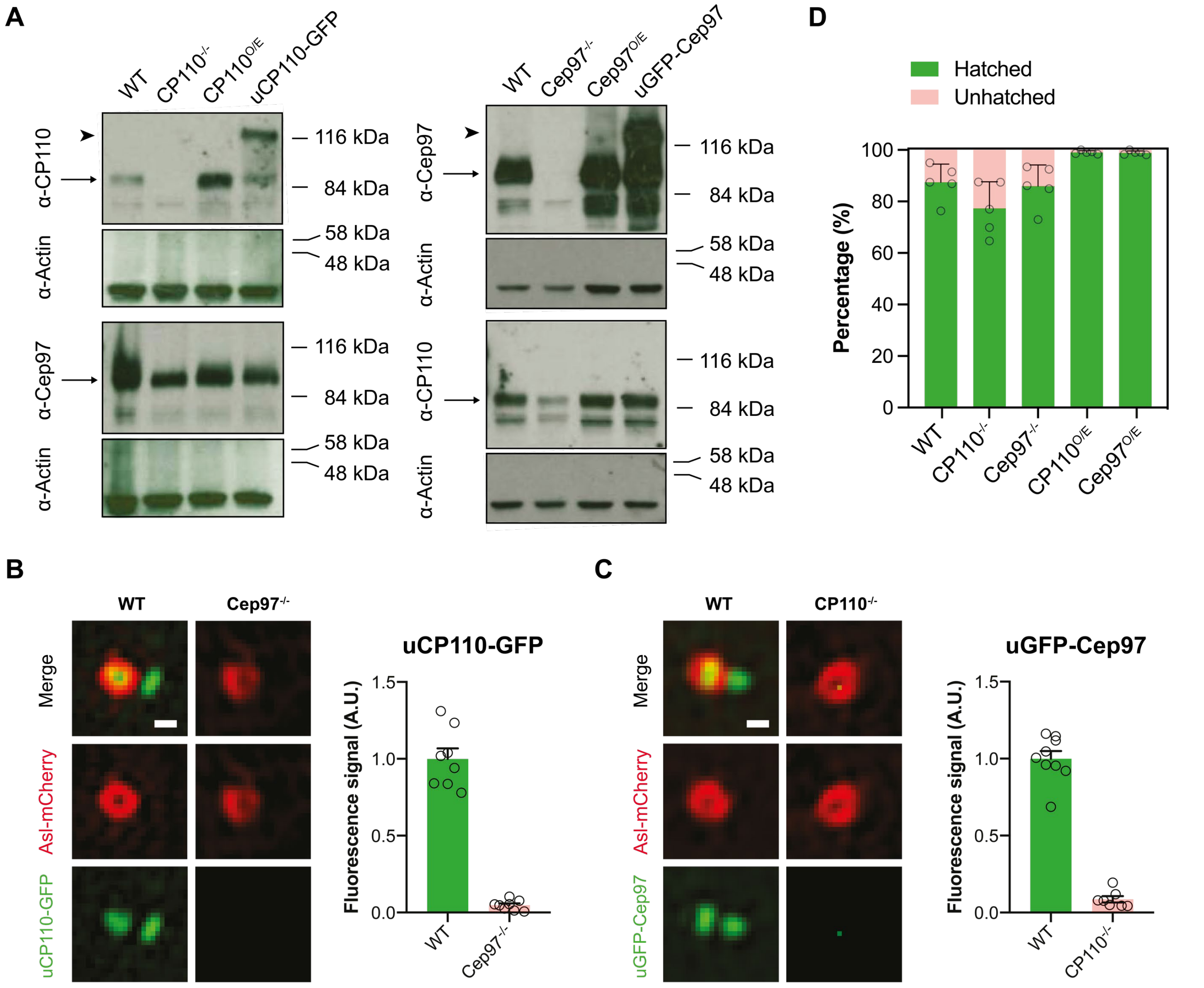

Fig. S1. CP110 and Cep97 are largely co-dependent for their centriolar localisation and partially co-dependent for their cytoplasmic stability. (A) Western blots show the protein levels of CP110 and Cep97 in WT embryos, *CP110* or *Cep97* null mutant embryos (-/-), embryos overexpressing untagged CP110 or Cep97 from the *Ubq* promoter (O/E), or embryos overexpressing CP110-GFP or GFP-Cep97 from the Ubq promoter (u). Actin is shown as a loading control. Representative blots are shown from three technical repeats. Note that there appears to be slightly less Cep97, which is less smeared, in the absence of CP110, while there is clearly less CP110 in the absence of Cep97. **(B and C)** Airy-scan micrographs shows the centriole localisation of either uCP110-GFP (*green*, B) in WT and *Cep97*$^{-/-}$ embryos or uGFP-Cep97 (*green*, C) in WT and *CP110*$^{-/-}$ embryos; Asl-mCherry (*red*) labels the mother centrioles (Scale bar=0.2 μm). Bar charts quantify the

centriolar levels (Mean±SD) of uCP110-GFP or uGFP-Cep97 in these embryos. For this quantification, the ten centriole pairs with the brightest Asl- mCherry were selected in each embryo (as CP110 or Cep97 levels could not be used to reliably identify the centrioles in the mutant embryos) and the centriolar levels of uCP110-GFP or uGFP-Cep97 was measured at 20 minutes into interphase of cycle 14. We analysed cycle 14 embryos in this experiment because CP110 and Cep97 centriolar levels rise to a steady plateau in the first 5-10mins of the extended interphase period in this cycle
(rather than dropping as the embryos prepare to enter mitosis as in the earlier cycles), so centriolar fluorescence is normally at a constantly high level at this stage. N≥7 embryos, n=10 centrioles per embryo. **(D)** Bar chart indicates the embryo hatching frequency in wild type flies (*Oregon R*) or in flies of the indicated genotypes.

Figure S2

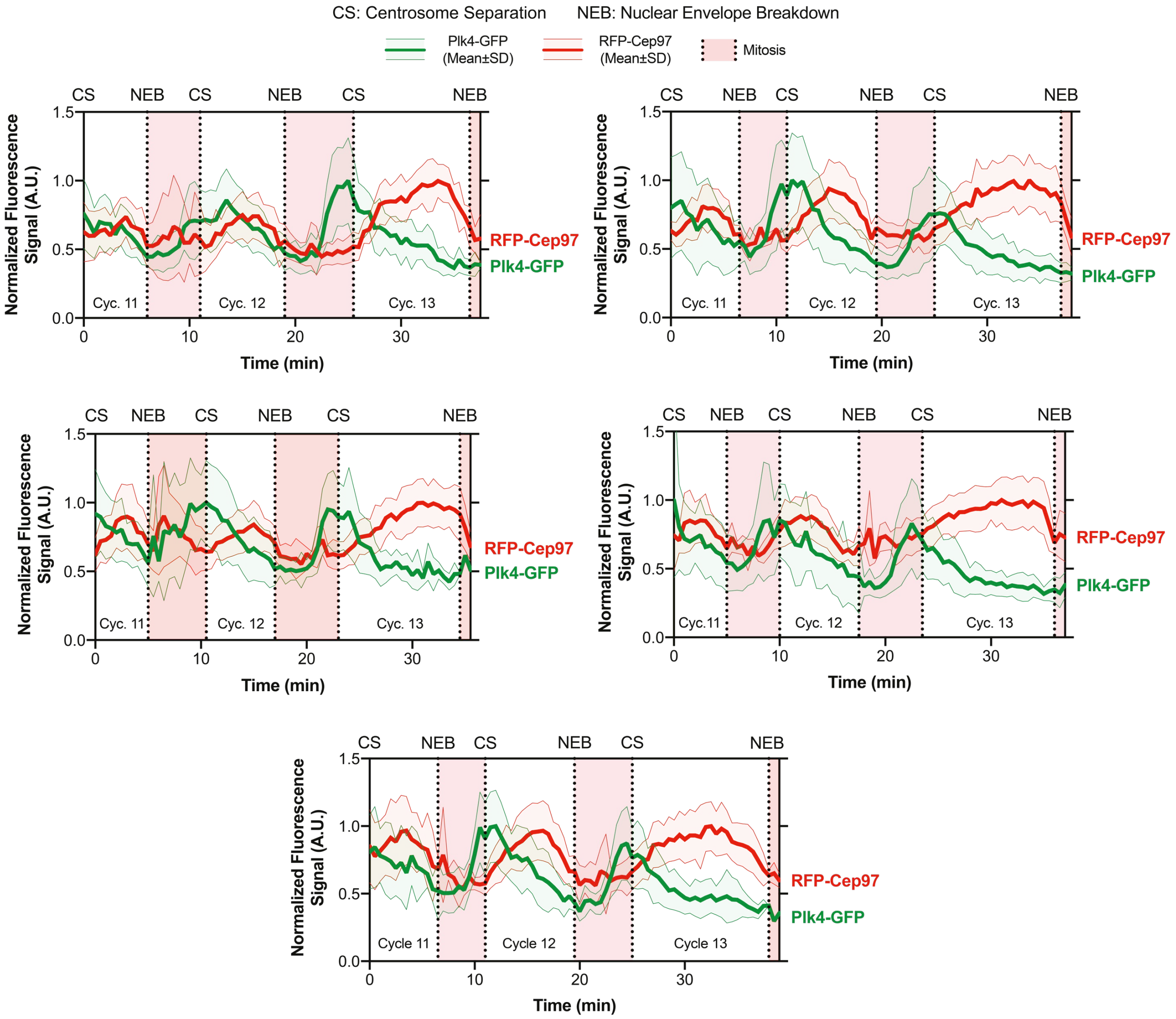

Fig. S2. The centriolar recruitment of Cep97 is largely out of phase with the centriolar recruitment of Plk4.

Graphs quantify the centriolar fluorescence levels (Mean±SD) of Plk4-GFP (*green*) and uRFP-Cep97 (*red*) co-expressed in five individual embryos analysed during nuclear cycles 11-13. CS= Centrosome Separation, NEB=Nuclear Envelope Breakdown. An average of n=41 centrioles were tracked per embryo.

Journal of Cell Science • Supplementary information

Figure S3

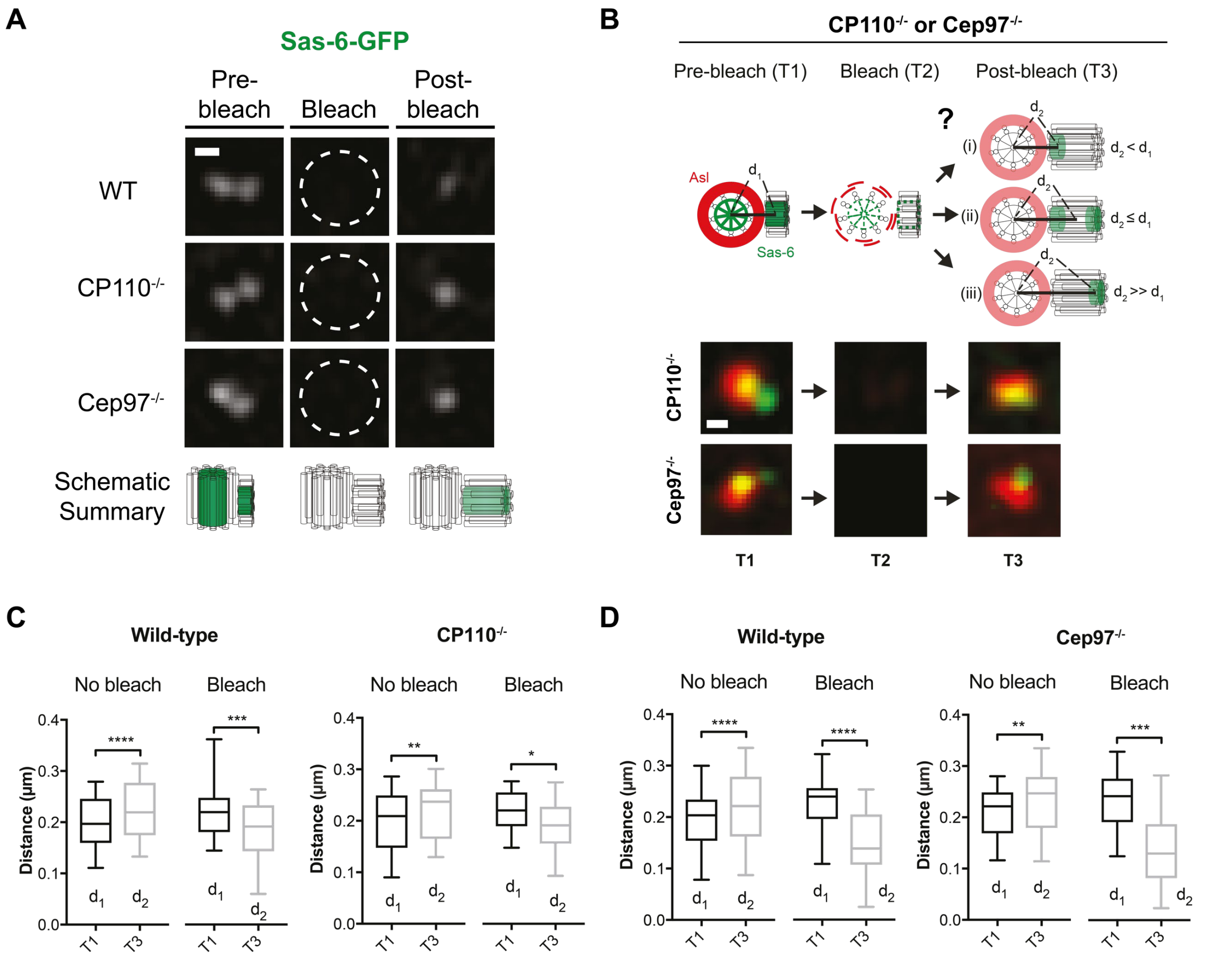

Fig. S3. The centriole cartwheel continues to grow preferentially from the proximal-end of daughter centrioles in CP110$^{-/-}$ and Cep97$^{-/-}$embryos.

(A) Micrographs show a 3D-SIM-FRAP analysis of Sas-6-GFP dynamics in WT, *CP110$^{-/-}$* and *Cep97$^{-/-}$* embryos. For each condition, a 3D-SIM image of a centriole pair was acquired in early/mid S-phase (Pre-bleach). The centrioles were subsequently photobleached (Bleach), and a 3D-SIM image was acquired 1 min after photobleaching (Post-bleach). These observations demonstrate that Sas-6-GFP continues to be incorporated exclusively into the growing daughter centriole even in the absence of CP110 or Cep97. Scale bar=0.2 µm. N≥8 embryos per group, n=3 centriole pairs on average per embryo. Schematics below each micrograph illustrate our interpretation of the FRAP experiments. **(B)** Schematic illustrates the photobleaching assay previously used to show that Sas-6-GFP preferentially incorporates into the proximal-end of growing daughter cartwheels (outcome [i]) (Aydogan et al., 2018). We used the same assay to test whether this was also the case in *CP110$^{-/-}$* and *Cep97$^{-/-}$* embryos. (Lower panel) Airy-scan super resolution micrographs show representative centriole images during pre-bleach (T1), bleach (T2) and post-bleach (T3) stages of the FRAP experiment in CP110$^{-/-}$

and Cep97$^{-/-}$ embryos simultaneously expressing Asl-mCherry and Sas-6- GFP. Scale bar=0.2 μm. **(C and D)** Box and whisker plots show the pre- and post-bleach distance (d_1 and d_2, respectively) between Asl-mCherry on the mother centriole and the newly incorporating Sas-6-GFP on the growing daughter centriole in *CP110$^{-/-}$* (C) or *Cep97$^{-/-}$* (D) embryos compared to WT controls. In the *No bleach* control experiment, $d_2 > d_1$ for all conditions, reflecting the growth of the daughter centriole between T1 and T3. In the *Bleach* experiment, $d_2 << d_1$ for all conditions, indicating that Sas-6-GFP continues to incorporate only into the proximal-end of the centrioles in the absence of CP110 or Cep97. N≥11 embryos per condition; n≥16 centriole pairs for *No Bleach* and *Bleach* groups each. Midlines represent the median, whiskers (error bars) mark the minimum to maximum, and bottom/top of the boxes indicate the first/third quartile of the distribution, respectively. Statistical significance was assessed using a paired *t* test (*, P<0.05; **, P<0.01; ***, P<0.001; ****, P<0.0001).

Journal of Cell Science • Supplementary information

Fig. S4. Blot transparency.
Uncropped western blots demonstrated in this paper.

In Figure 4

CP110 and Actin blot:

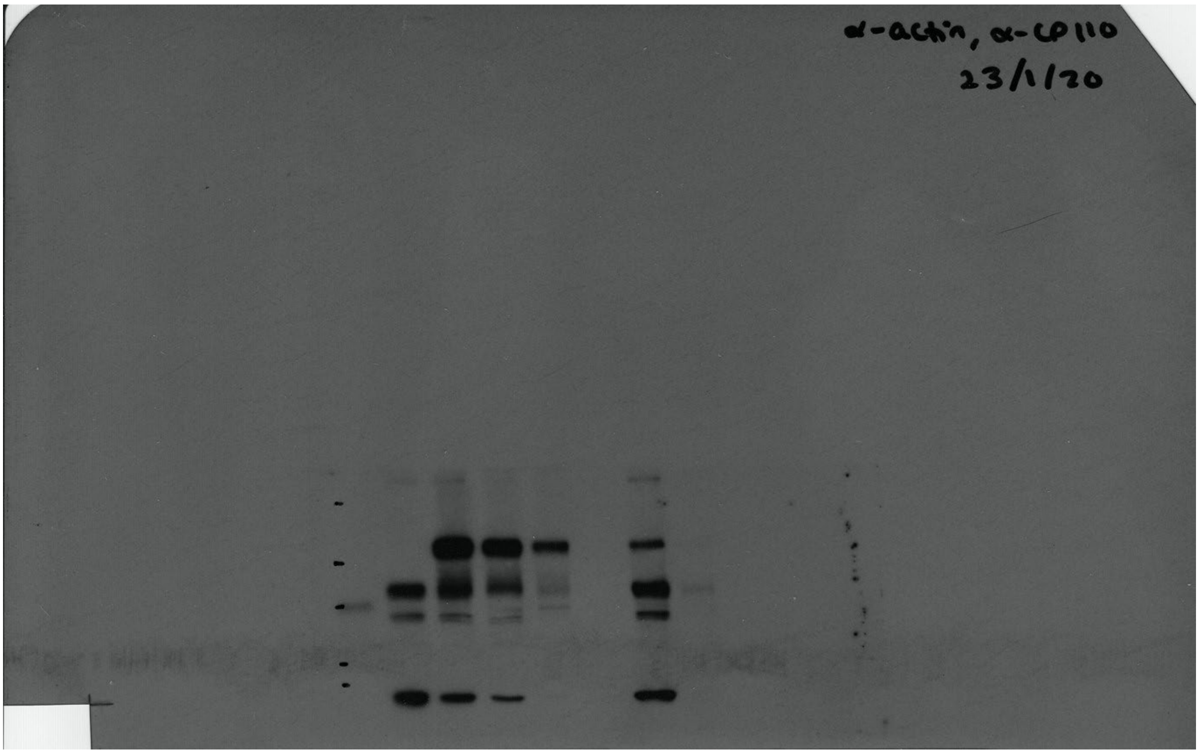

CP110 and Actin blot with explanations:

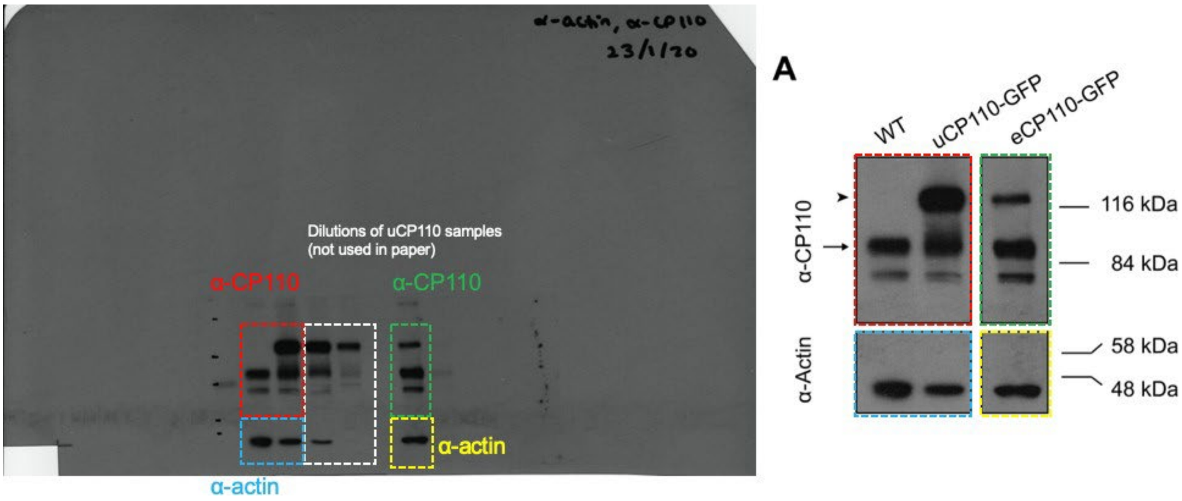

Journal of Cell Science • Supplementary information

Cep97 blot:

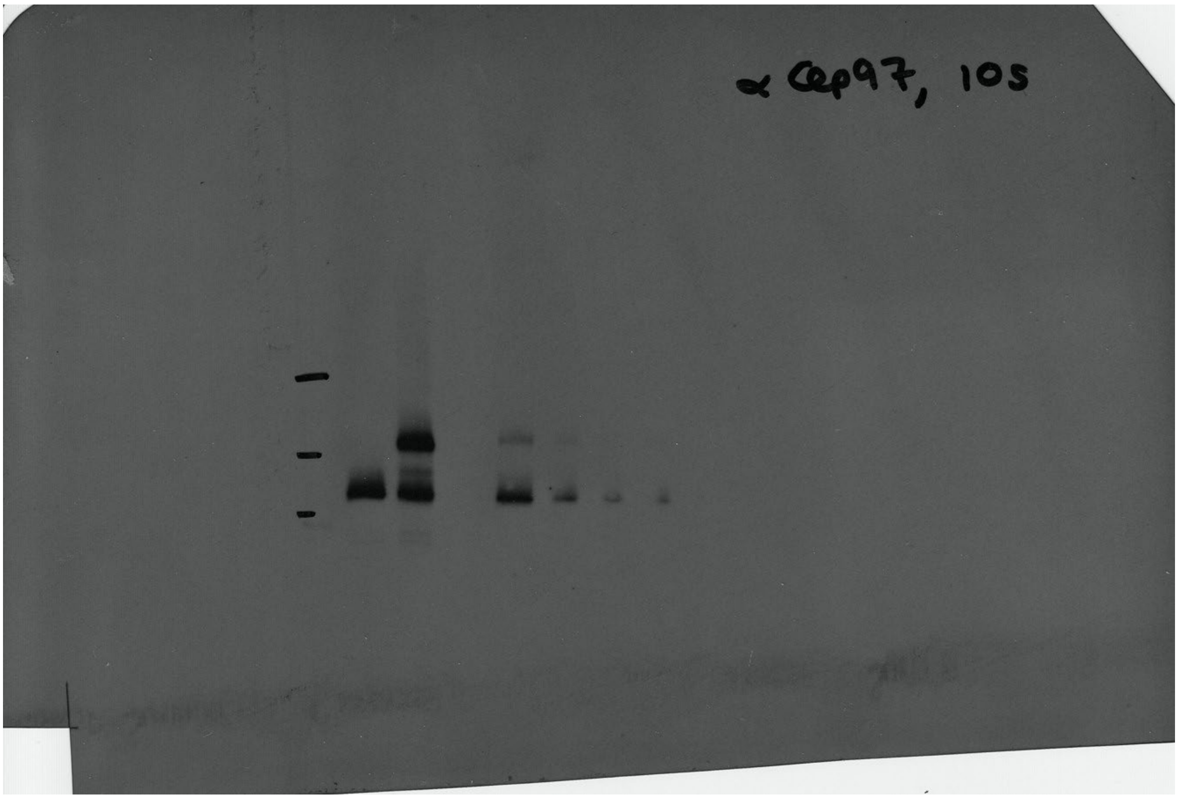

CNN blot:

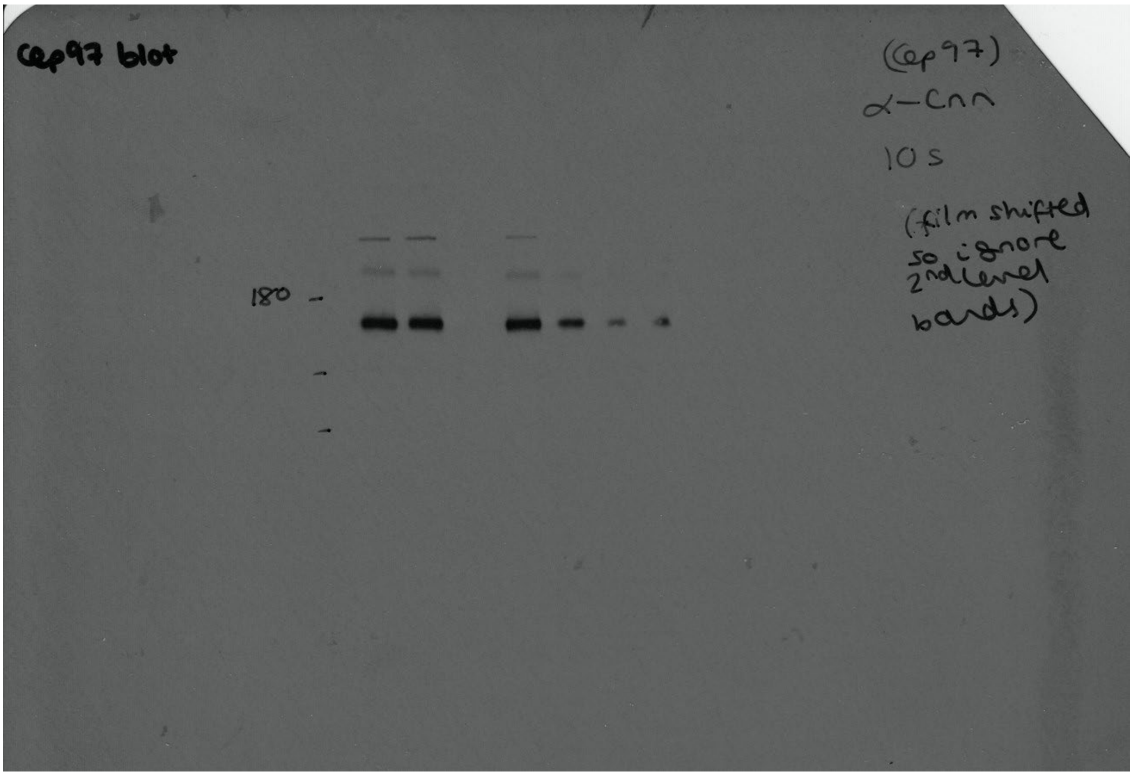

Journal of Cell Science • Supplementary information

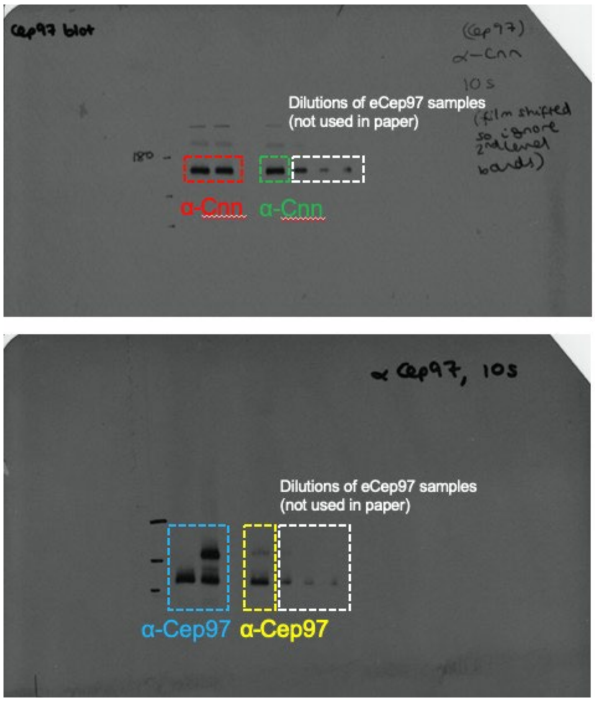

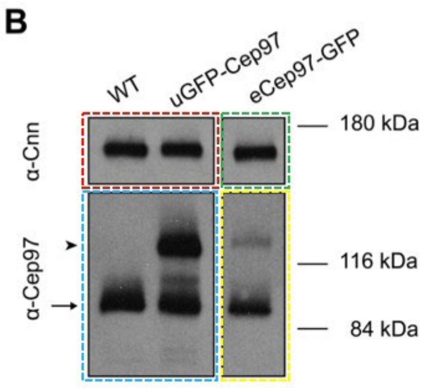

Journal of Cell Science • Supplementary information

CP110 blot:

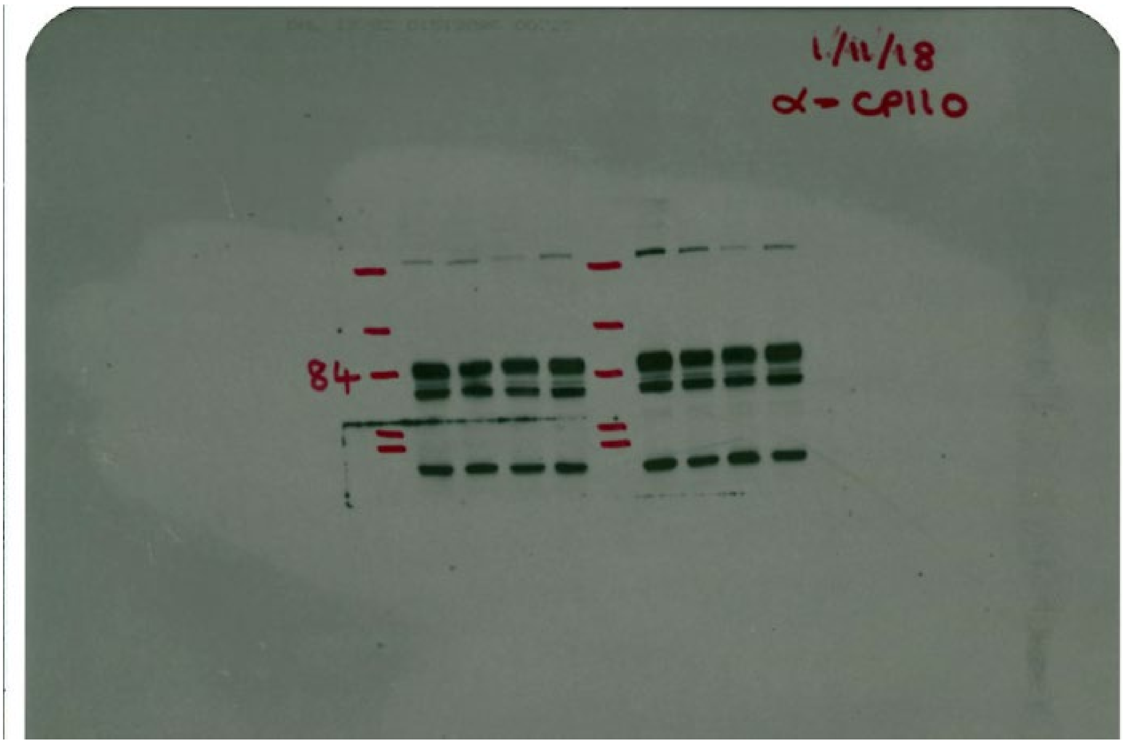

CP110 blot with explanations:

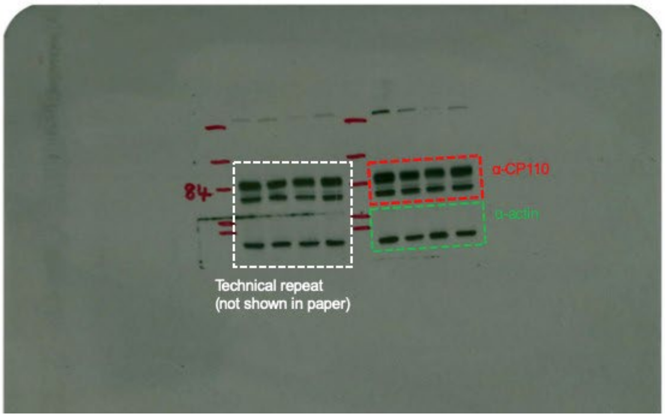

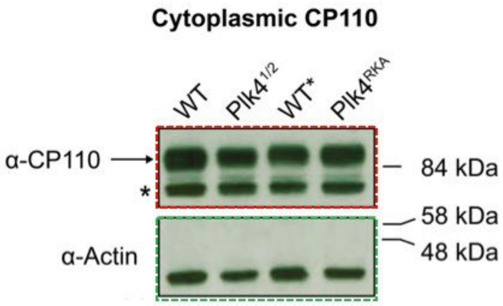

Journal of Cell Science • Supplementary information

Cep97 blot:

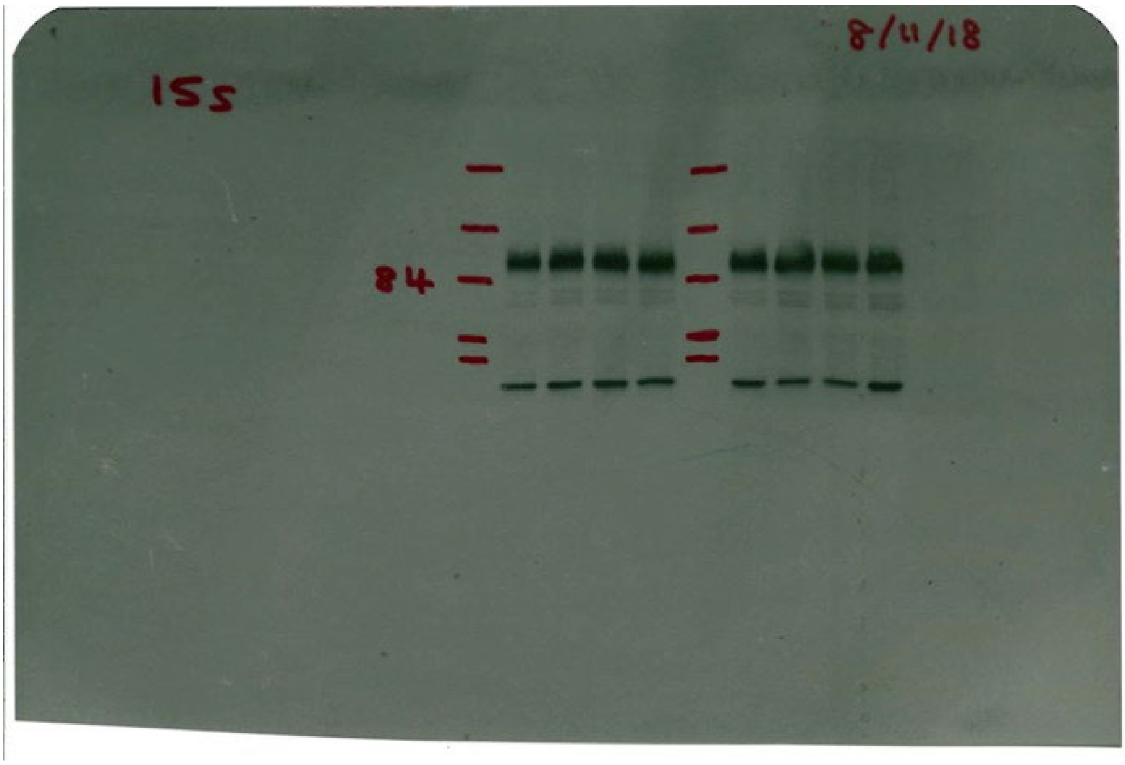

Cep97 blot with explanations:

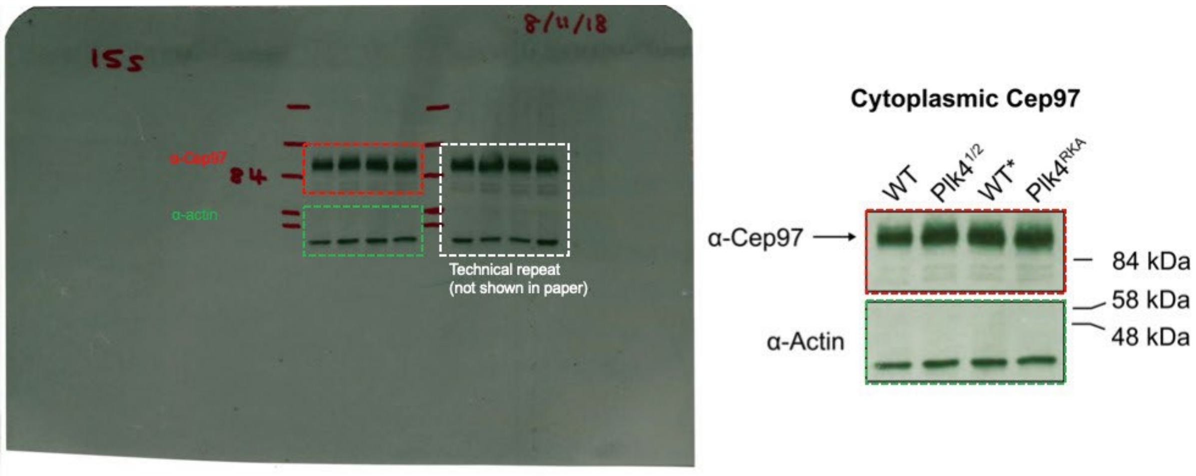

CP110 and Cep97 blots (left panels):

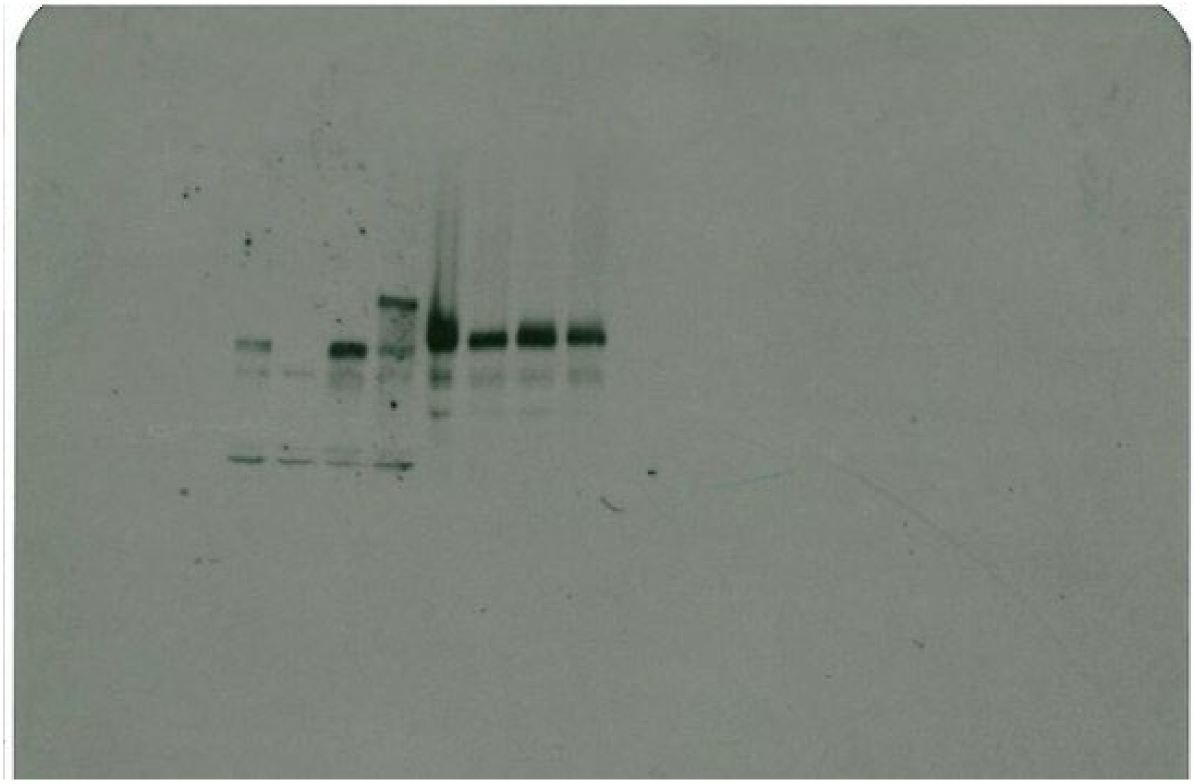

CP110 and Cep97 blots with explanations (left panels):

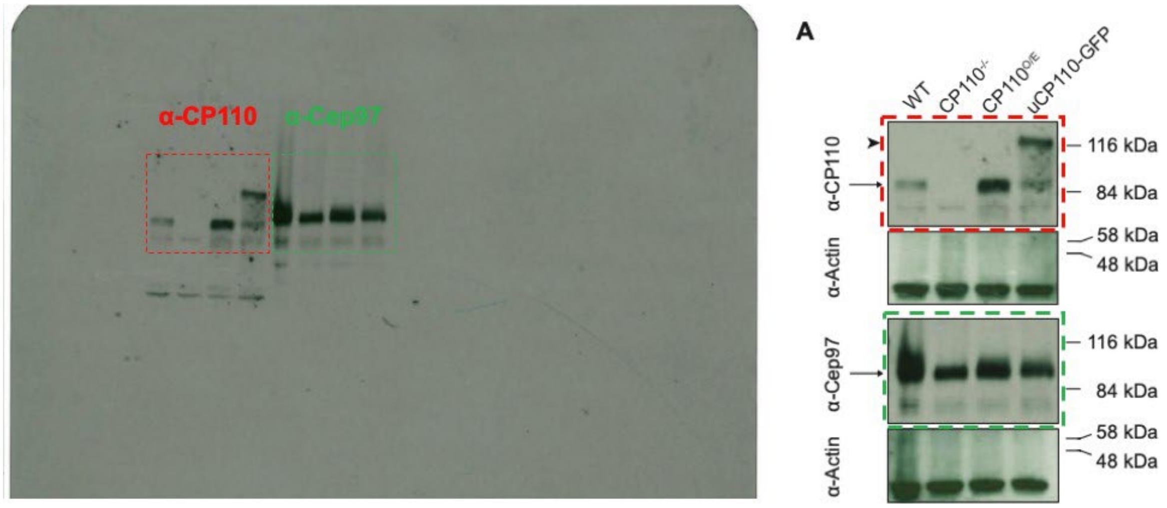

Journal of Cell Science • Supplementary information

Actin blot (left panels):

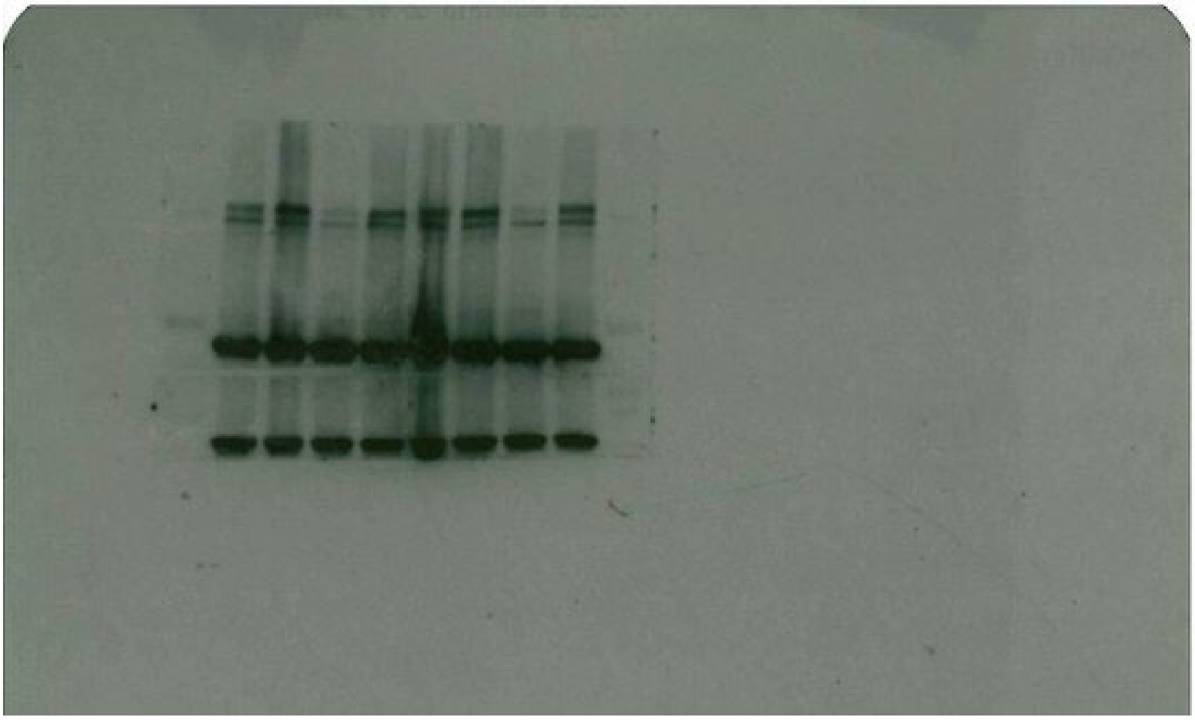

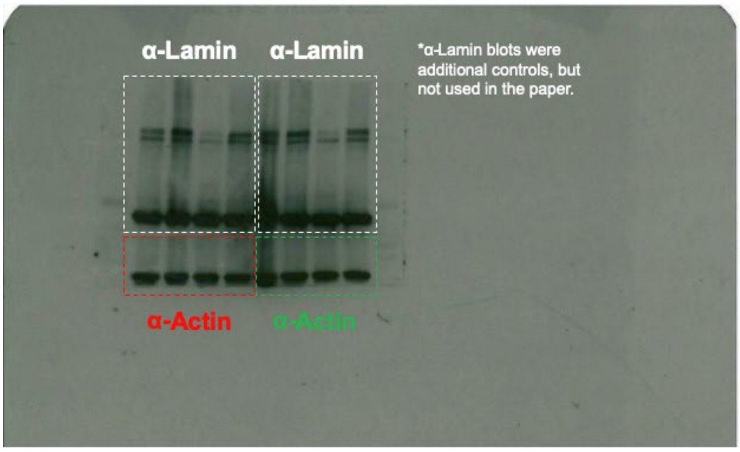

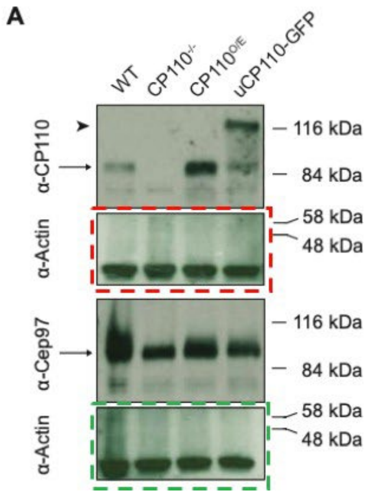

Journal of Cell Science • Supplementary information

CP110 and Cep97 blots (right panels):

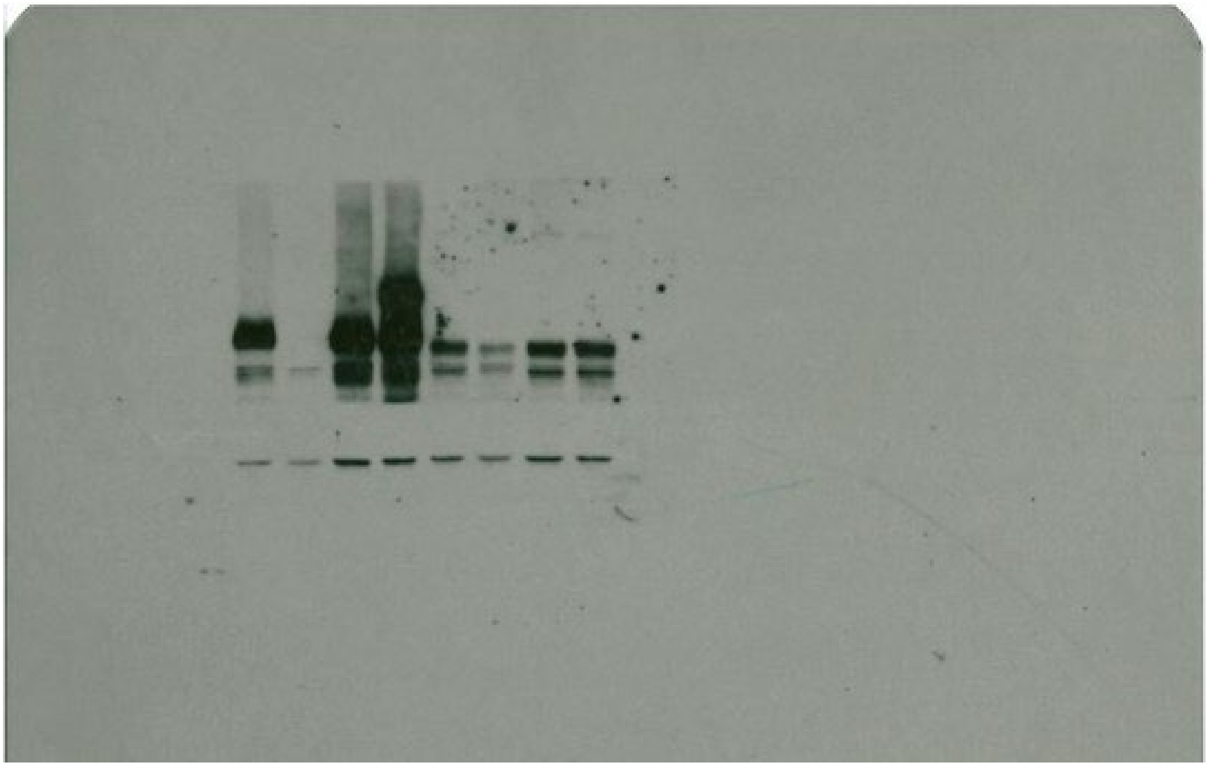

CP110 and Cep97 blots with explanations (right panels):

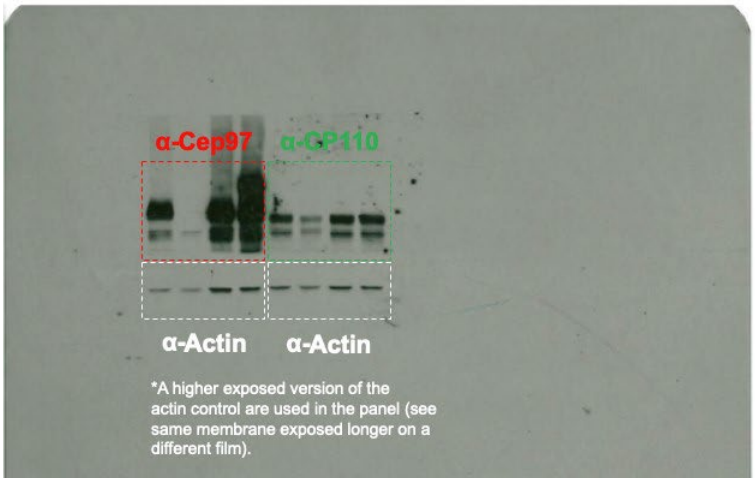

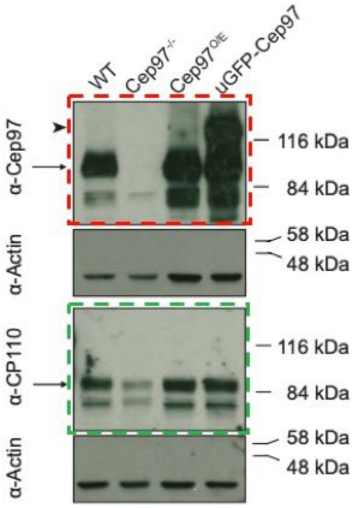

Journal of Cell Science • Supplementary information

Actin blot (right panels):

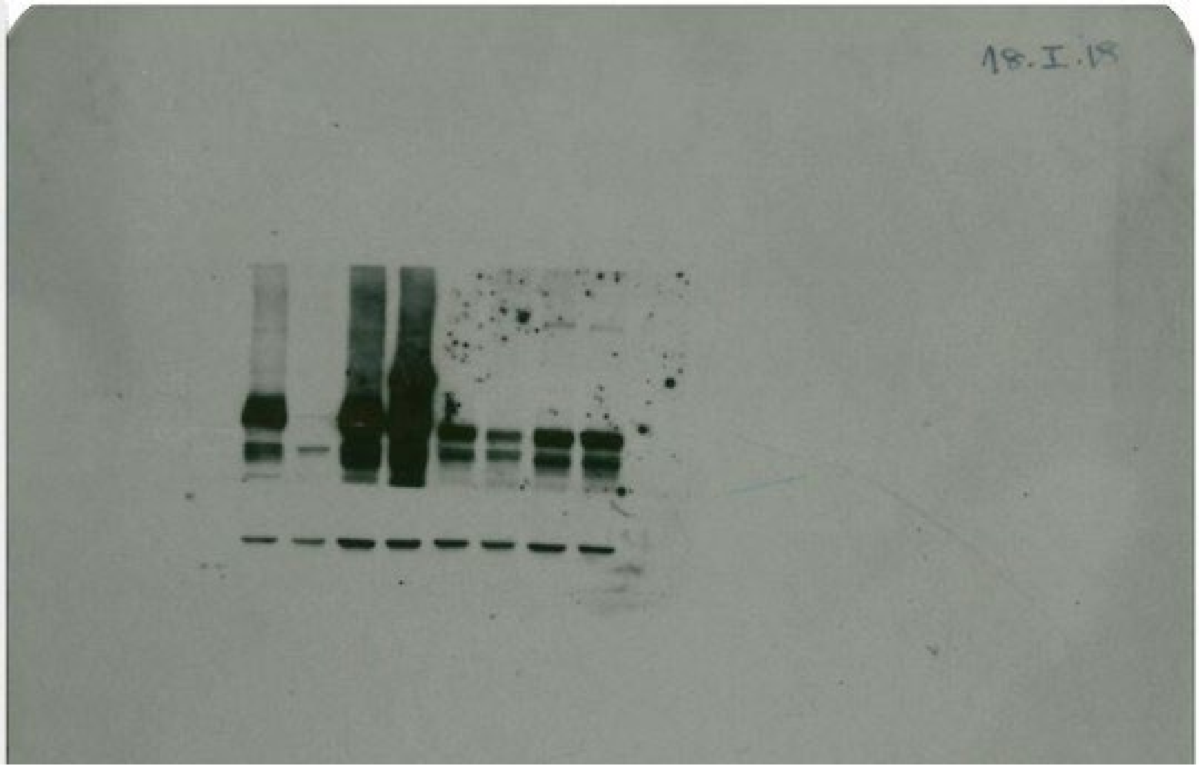

Actin blot with explanations (right panels):

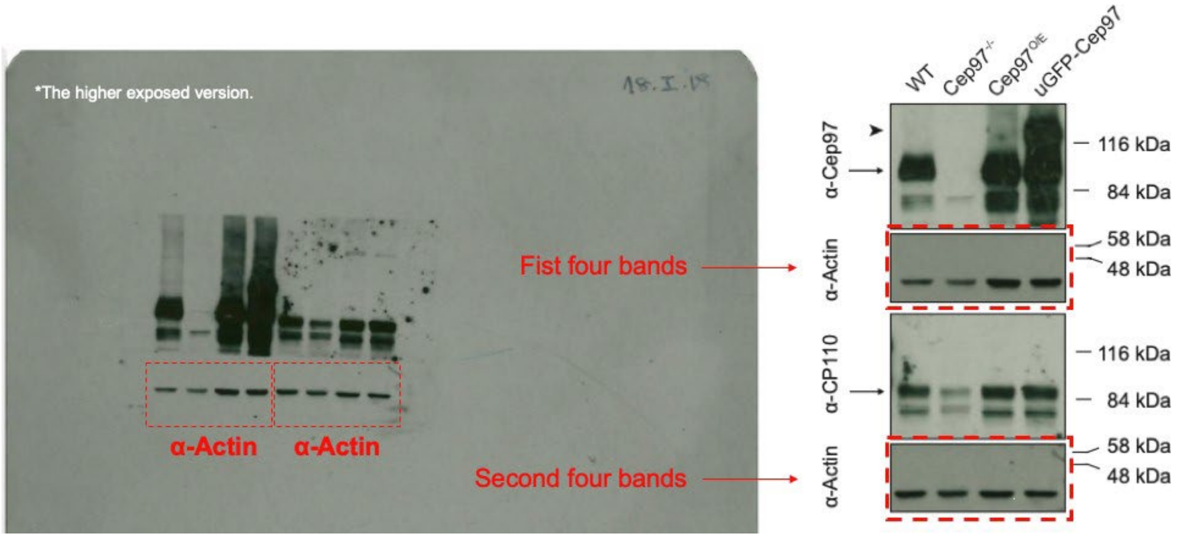

Journal of Cell Science • Supplementary information

Table S1. *D. melanogaster* alleles used in this study.

Allele*	Source (reference #)	ID
Ubq-GFP-Cep97	(Dobbelaere et al., 2008)	FlyBase ID: FBal0343980
Ubq-CP110-GFP	(Dobbelaere et al., 2008)	FlyBase ID: FBtp0092320
Ubq-CP110	This paper	N/A
Ubq-Cep97	This paper	N/A
Cep97-GFP	(Dobbelaere et al., 2020)	FlyBase ID: FBal0362412
CP110-GFP	This paper	N/A
Plk4-GFP	(Aydogan et al., 2018)	FlyBase ID: FBal0343977
Ubq-RFP-Cep97	(Dobbelaere et al., 2008)	N/A
$Plk4^{Aa74}$	(Aydogan et al., 2018)	FlyBase ID: FBab0049012
CP110Δ	(Franz et al., 2013)	FlyBase ID: FBal0294119
Cep97Δ	(Dobbelaere et al., 2020)	FlyBase ID: FBal0362411
Asl-mCherry	(Conduit et al., 2015)	FlyBase ID: FBal0343645
Ubq-Cep97-GFP	(Dobbelaere et al., 2008)	N/A
Sas-6-GFP	(Aydogan et al., 2018)	FlyBase ID: FBtp0131375
Plk4	(Aydogan et al., 2018)	FlyBase ID: FBal0343978
$Plk4^{RKA}$	(Aydogan et al., 2018)	FlyBase ID: FBtp0131379
Asl-mKate2	(Aydogan et al., 2020)	FlyBase ID: FBal0366991
asl^{B46}	(Baumbach et al., 2015)	FlyBase ID: FBal0343439
$CycB^{2}$	(Jacobs et al., 1998)	FlyBase ID: FBal0094855

*The alleles listed here were expressed under their endogenous promoters unless otherwise specified.

Journal of Cell Science • Supplementary information

Table S2. *D. melanogaster* strains generated and/or used in this study.

Strain Genotype	Tissue	Type of experiment
Ubq-GFP-Cep97 / +	Embryo	Confocal microscopy; Western blot; Fluorescence correlation spectroscopy
Ubq-CP110-GFP / +	Embryo	Confocal microscopy; Western blot; Fluorescence correlation spectroscopy
Cep97-GFP / +	Embryo	Confocal microscopy; Western blot
CP110-GFP / +	Embryo	Confocal microscopy; Western blot
Ubq-CP110-GFP / +; $Plk4^{Aa74}$ / +	Embryo	Fluorescence correlation spectroscopy
Ubq-GFP-Cep97/ +; +/+; $Plk4^{Aa74}$ / +	Embryo	Fluorescence correlation spectroscopy
Plk4-GFP / +	Embryo	Confocal microscopy; Peak counting spectroscopy
CP110Δ / CP110Δ; Plk4-GFP / +	Embryo	Confocal microscopy; Peak counting spectroscopy
Plk4-GFP / Ubq-CP110	Embryo	Confocal microscopy; Peak counting spectroscopy
Plk4-GFP, Cep97Δ / Cep97Δ	Embryo	Confocal microscopy; Peak counting spectroscopy
Plk4-GFP / Ubq-Cep97	Embryo	Confocal microscopy; Peak counting spectroscopy
Asl-mCherry / Ubq-CP110-GFP	Embryo; wing discs	3D-SIM; Airy-scan super resolution microscopy
Ubq-GFP-Cep97 / +; Asl-mCherry / +	Embryo; wing discs	3D-SIM; Airy-scan super resolution microscopy
Asl-mCherry / +; Ubq-Cep97-GFP / +	Wing discs	3D-SIM

Journal of Cell Science • Supplementary information

Table S2 Cont'd: *D. melanogaster* strains generated and/or used in this study.

**Strain Genotype**	**Tissue**	**Type of experiment**
Oregon-R (Wild-type strain)	Embryo	Western Blot; Hatching assay; Fluorescence correlation spectroscopy
CP110Δ / CP110Δ	Embryo	Western blot; Hatching assay
Ubq-CP110 / +	Embryo	Western blot; Hatching assay
Cep97Δ / Cep97Δ	Embryo	Western blot; Hatching assay
Ubq-Cep97 / +	Embryo	Western blot; Hatching assay
Ubq-CP110-GFP / Ubq-RFP-Cep97	Embryo	Confocal microscopy
Sas-6-GFP / +	Embryo	Confocal microscopy; Airy-scan super resolution microscopy
CP110Δ / CP110Δ; Sas-6-GFP / +	Embryo	Confocal microscopy; Airy-scan super resolution microscopy
Sas-6-GFP / Ubq-CP110	Embryo	Confocal microscopy
Sas-6-GFP, Cep97Δ / Cep97Δ	Embryo	Confocal microscopy; Airy-scan super resolution microscopy
Sas-6-GFP / Ubq-Cep97	Embryo	Confocal microscopy
Asl-mCherry / Sas-6-GFP	Embryo	Airy-scan super resolution microscopy
CP110Δ / CP110Δ; Asl-mCherry / Sas-6-GFP	Embryo	Airy-scan super resolution microscopy
Asl-mCherry, Cep97Δ / Sas-6-GFP, Cep97Δ	Embryo	Airy-scan super resolution microscopy
Ubq-GFP-Cep97 / +; Plk4 /+	Embryo	Fluorescence correlation spectroscopy

Journal of Cell Science • Supplementary information

Table S2 Cont'd: *D. melanogaster* strains generated and/or used in this study.

Strain Genotype	Tissue	Type of experiment
Ubq-GFP-Cep97 / +; + /+; $Plk4^{RKA}$ / +	Embryo	Fluorescence correlation spectroscopy
Ubq-CP110-GFP / Plk4	Embryo	Fluorescence correlation spectroscopy
Ubq-CP110-GFP / +; $Plk4^{RKA}$ / +	Embryo	Fluorescence correlation spectroscopy
$Plk4^{Aa74}$ / +	Embryo	Western blot
Plk4 / +	Embryo	Western Blot
$Plk4^{RKA}$ / +	Embryo	Western blot
Asl-mKate2, asl^{B46} / +	Embryo	Peak counting spectroscopy
Plk4-GFP / Ubq-RFP-Cep97	Embryo	Confocal microscopy
Ubq-CP110-GFP / $CycB^{2}$	Embryo	Confocal microscopy
Ubq-GFP-Cep97 / +; $CycB^{2}$ / +	Embryo	Confocal microscopy

Journal of Cell Science • Supplementary information

Table S3. Oligonucleotides used in this study.

Oligonucleotide name	Sequence	Source
Primer to introduce a stop codon into pDONR-CP110: **Forward**	CAAACATCGCCGATTGGATTAGGACCCAGCTTTCTTGTAC	Invitrogen, Thermo Fisher Scientific
Primer to introduce a stop codon into pDONR-CP110: **Reverse**	GTACAAGAAAGCTGGGTCCTAATCCAATCGGCGATGTTTG	Invitrogen, Thermo Fisher Scientific
Primer to clone the C-terminal fragment aa 329-807 of Cep97 into the pDONR vector: **Forward**	GGGGACAAGTTTGTACAAAAAAGCAGGCTTGTTCTCCCGCTTGAGTGGCCGCCAGG	Invitrogen, Thermo Fisher Scientific
Primer to clone the C-terminal fragment aa 329-807 of Cep97 into the pDONR vector: **Reverse**	GGGGACCACTTTGTACAAGAAAGCTGGGTGTCATGGATCTTTATCAAGATTTTC	Invitrogen, Thermo Fisher Scientific
Primer to amplify the cp110 promoter region: **Forward**	TGTACAAAAAAGCAGGCTTCGTTCCCTTTCGCTGTCAAG	Invitrogen, Thermo Fisher Scientific
Primer to amplify the cp110 promoter region: **Reverse**	ATTGCCCACGTCGCATCCATTGGTGTTTTGCTACTGGG	Invitrogen, Thermo Fisher Scientific
Primer to amplify the pDONR-Zeo vector containing the *cp110* sequence: **Forward**	ATGGATGCGACGTGGGCA	Invitrogen, Thermo Fisher Scientific
Primer to amplify the pDONR-Zeo vector containing the *cp110* sequence: **Reverse**	GAAGCCTGCTTTTTTGTAC	Invitrogen, Thermo Fisher Scientific

Journal of Cell Science • Supplementary information

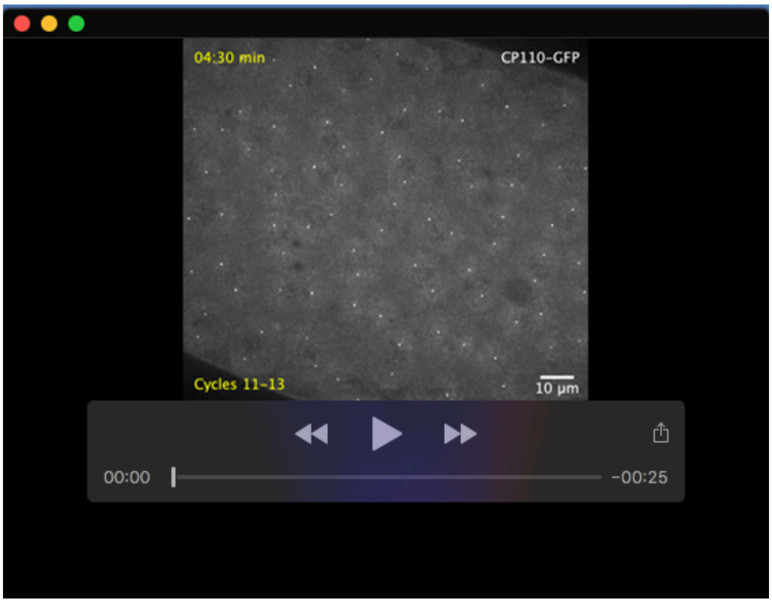

Movie 1. Monitoring the centriolar dynamics of uCP110-GFP in a *Drosophila* embryo. Time-lapse video of an embryo expressing uCP110-GFP, observed on a spinning-disk confocal microscope through nuclear cycles 11-13. The movie is a maximum-intensity projection that has been photo-bleach corrected, but not background subtracted for visual clarity. Time (min:sec) is shown at the top left, and the developmental stage of the embryo is indicated at the bottom left.

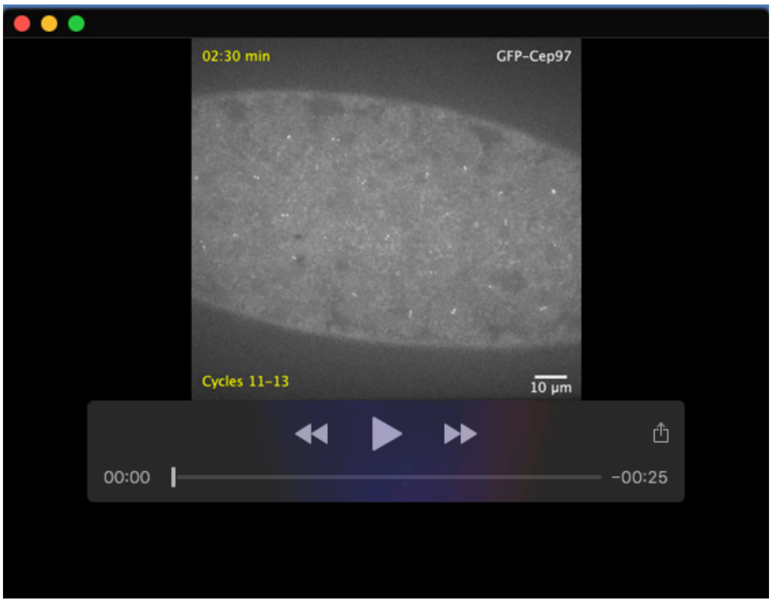

Movie 2. Monitoring the centriolar dynamics of uGFP-Cep97 in a *Drosophila* embryo. Time-lapse video of an embryo expressing uGFP-Cep97, observed on a spinning-disk confocal microscope through nuclear cycles 11-13. The movie is a maximum-intensity projection that has been photo-bleach corrected, but not background subtracted for visual clarity. Time (min:sec) is shown at the top left, and the developmental stage of the embryo is indicated at the bottom left.

Journal of Cell Science • Supplementary information

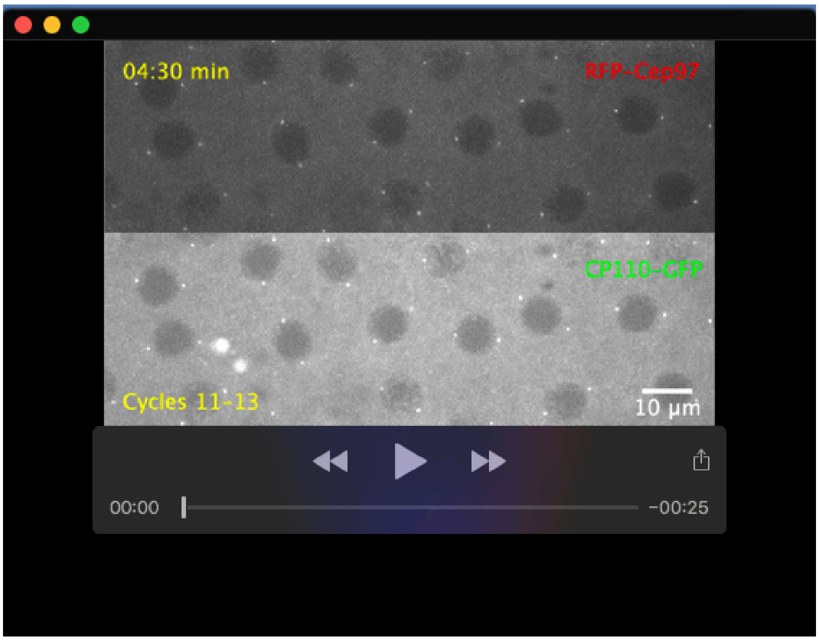

Movie 3. Monitoring the centriolar dynamics of uCP110-GFP and uRFP-Cep97 simultaneously in the same embryo.
Time-lapse movie of an embryo expressing uCP110-GFP and uRFP-Cep97, observed on a spinning-disk confocal microscope through nuclear cycles 11-13. The movie is a maximum-intensity projection that has been photo-bleach corrected, but not background subtracted for visual clarity. Time (Min:Sec) is shown at the top left, and the developmental stage of the embryo is indicated at the bottom left.

References not in main text

Baumbach, J., Novak, Z. A., Raff, J. W. and Wainman, A. (2015). Dissecting the function and assembly of acentriolar microtubule organizing centers in Drosophila cells in vivo. *PLoS Genet.* **11**, e1005261. doi:10.1371/journal.pgen.1005261

Conduit, P. T., Wainman, A., Novak, Z. A., Weil, T. T. and Raff, J. W. (2015). Reexamining the role of Drosophila Sas-4 in centrosome assembly using two-colour-3D-SIM FRAP. *ELife* **4**, e08483. doi:10.7554/eLife.08483

Jacobs, H. W., Knoblich, J. A. and Lehner, C. F. (1998). Drosophila Cyclin B3 is required for female fertility and is dispensable for mitosis like Cyclin B. *Genes Dev.* **12**, 3741-3751. doi:10.1101/gad.12.23.3741
