# Supplementary material for: Centriole distal-end proteins CP110 and Cep97 influence centriole cartwheel growth at the proximal end
Source: J Cell Sci. 2022 Jul 20;135(14):jcs260015. doi: 10.1242/jcs.260015 (PMC9450887; doi:10.1242/jcs.260015)
Supplement: Reviewer comments [file joces-135-260015_review_history.pdf]

Centriole distal-end proteins CP110 and Cep97 influence centriole cartwheel growth at the proximal end

Mustafa G. Aydogan, Laura E. Hankins, Thomas L. Steinacker, Mohammad Mofatteh, Saroj Saurya, Alan Wainman, Siu-Shing Wong, Xin Lu, Felix Y. Zhou and Jordan W. Raff
DOI: 10.1242/jcs.260015

Editor: David Stephens

Review timeline

Submission to Review Commons:	18 Aug 2021
Original submission:	14 March 2022
Editorial decision:	8 April 2022
First revision received:	9 June 2022
Accepted:	13 June 2022

Reviewer 1

Evidence, reproducibility and clarity

This manuscript is a continuation of the previous articles of the authors (Aydogan et al., JCB 217:1233, 2018; Aydogan et al., Cell 181:1566, 2020). They reported that Plk4 initiates and times the growth of the cartwheel at the proximal end during early divisions of the Drosophila embryos. In this manuscript, they investigated roles of the CP110/Cep97 complex in the centriole growth control at the distal end of the centriole. The daughter centriole levels of the CP110/Cep97 complex oscillate in S phase in a similar manner to those of Plk4. The CP110/Cep97 oscillation is entrained by the core Cdk/Cyclin cell cycle oscillator but not by Plk4. Rather, the centriolar levels of Plk4 increased in the CP110 and Cep97 deletion embryos. The experiments seem to be carefully carried out, data are nicely presented, and manuscript is clearly written.

Significance

I agree with their interpretation that the CP110/Cep97 oscillation does not appear to play a major part in determining the period of daughter centriole growth during early divisions of the Drosophila embryos. The CP110/Cep97 complex seems to have a limited role in the centriole length control. The CP110/Cep97 complex may be important to prevent centrioles from over-elongating after the initial growth of centrioles.

As suggested in the manuscript, phosphorylation may be a regulatory mechanism for CP110 behaviors at the centrioles. It was previously reported that CP110 is a substrate of the cell cycle kinases, such as Cdk2 (Chen et al., Dev Cell 3:339, 2002) and Plk4 (Lee et al., Cell Cycle 16:1225, 2017).
Phosphorylation may be required for recruitment or removal of CP110 at the centrioles. Nonetheless, it is hard to interpret the functional significance of the S phase oscillation of the CP110/Cep97 complex with the data in the manuscript.

It is unfortunate to conclude that the CP110/Cep97 complex may not be a major player for controlling the centriole growth. However, the manuscript includes other interesting observations. For example, they presented data supporting that the SAS6 protein is added at the proximal side of the centrioles, which is opposite to the microtubule growth.
Microtubules in the daughter centrioles may assemble at the minus end rather than the plus end. It would be interesting to determine when γ-tubulins are recruited to the growing centrioles.

© 2022. Published by The Company of Biologists under the terms of the Creative Commons Attribution License (https://creativecommons.org/licenses/by/4.0/).

Reviewer 2

Evidence, reproducibility and clarity

In this study, Aydogan, Hankins, and colleagues, present an interesting work that follows up on their article "An Autonomous Oscillation Times and Executes Centriole Biogenesis" published last year in Cell. In this new study, they analyzed the distal complex consisting of CP110/Cep97 in the centriole of Drosophila embryos. They first demonstrated their oscillatory recruitment at the distal tip of the daughter centriole and they proposed that this protein complex is implicated in the control of centriole growth timing. They also demonstrated the importance of the crosstalk between CP110/Cep97 and Plk4 and its impact on cartwheel growth. This paper proposes a compelling model explaining how centriole growth is regulated. This manuscript is very well written and the data is of high quality. However, some point needs to be clarified before publication:

Major points:

- Figure 1: Since SAS-4 and CP110/CEP97 are only 5nm apart, SAS-4/CPAP is thought to have an antagonistic function to CP110 in the regulation of centriolar growth, and Plk4 can phosphorylate CPAP (DOI: 10.1038/emboj.2010.118), do the authors think that SAS-4 might also be involved in cartwheel/centriole elongation? Does SAS-4 oscillate?
- Figure S1B: The reduction in the intensity of CP110 in Cep97-/- and of Cep97 in CP110-/- is very obvious, nevertheless it is surprising that the cytoplasmic background, even reduced, is not visible, the images are completely dark. Would it be possible to image with a higher laser power or boost the intensity to see if a small amount is present at centrioles?
- Figure 3: The authors indicate that "uGFP-CP110 or uGFP-CEP97 levels remained relatively constant on the mother". However, the intensity clearly decreases over time. Can the authors explain this result, is it due to photobleaching?
- Do the oscillations of CP110 and Cep97 occur at or around the tip of the growing centriole? Would it be possible to use super-resolution at different stages of the S-phase to answer this question?
- The authors indicate that the level of overexpression of CP110-GFP and Cep97-GFP is 2.5X compared to their endogenous proteins (based on the western blot in Figure S1). Nevertheless, it seems that the overexpression of CP110 is more important. Quantification is necessary here.
- The authors proposed that "The CP110/Cep97 oscillation is entrained by the Cdk/Cyclin cell cycle" because they observed a strong and significant correlation between the timing of the CP110/Cep97 peak and S-phase length for both uGFP-Cep97 and uCP110-GFP at all nuclear cycles. It seems to me that this correlation is not sufficient for this statement. If it is not possible to inhibit the CCO to check its impact on CP110/Cep97, this statement should be mitigated.
- Figure 6: According to your results, cartwheels are longer in absence of CP110 or CEP97 and opposite in overexpression situations. Does the intensity perfectly reflect the length of the cartwheel? is the centriole longer? Could you confirm your observation on cartwheel/centriole length using electron microscopy?

Minor points:

- Figure 1C: as the authors show that CP110 and Cep97 are localizing at the distal end of the centriole, I suggest that they place CP110 and Cep97 distally and not at the level of the cartwheel, this representation can be misleading and suggest that CP110 and Cep97 are part of the cartwheel/MT connection.

Significance

The results presented are new and quite unexpected. This work allows a better understanding of phenotypes previously observed. I believe that this work will have an important impact in the field as it brings a whole new vision on the regulation of centriole growth.
This article is primarily aimed at centriole/centrosome/cilia fields but may be of interest to a broader cell biology audience.

My field of expertise is centriole/cilia biology

© 2022. Published by The Company of Biologists under the terms of the Creative Commons Attribution License (https://creativecommons.org/licenses/by/4.0/).

Reviewer 3

Evidence, reproducibility and clarity

SUMMARY

This study uses nuclear cycles 11-13 of Drosophila embryos to show the dynamics of the distal centriole localizing CP110/Cep97 complex during the predicted time of MT assembly during new centriole assembly. Continuing from prior work from this group, the authors find that the increase and decrease in CP110/Cep97 at new centrioles correlates with the timing of Cdk/Cyclin oscillations (CCO). The authors find that increased or decreased levels of CP110/Cep97 changes the dynamics of SAS6 and Plk4 levels. The authors suggest that there is crosstalk between the distal localizing CP110/Cep97 complex and the proximal localizing Plk4 and SAS6 proteins required for early centriole assembly.

MAJOR

Overall, the results are potentially interesting but I believe that there a number of instances in this manuscript where the conclusions need to either be strengthened with further experiments or toned down to reveal exactly what is shown in the manuscript.

CP110/Cep97 OSCILLATIONS

Because oscillations are repetitive variation in levels/activity with time, I think the manuscript needs to either use other terms that accurately describe what is measuring here or it should be defined what the authors are calling an oscillation. CP110/Cep97 only increases and then decreases during a single new centriole assembly and maturation event and I think that this should be clearly describe it this way.

LOCALIZATION OF CP110/Cep97 TO DISTAL END OF CENTRIOLES

Based on the existing published studies, it is clear that CP110/Cep97 localizes to the distal end of centrioles. Figure 1 does not show distal centriole localization in daughter centrioles of the syncytium that are the subject of this manuscript though. Its shows radial localization in the mother centriole of the fly wing. Figure 1 therefore has not relevance to the rest of the manuscript and has already been shown in prior studies.

My suggestion would be that this figure should study the dynamic localization of CP110/Cep97 at daughter centrioles during new centriole assembly in the syncytium. Moreover, this should localize these proteins relative to SAS6 and Plk4 that are the subject of the manuscript.
Are there localization dynamic changes during the oscillation? Are there times when these proteins do co-localize?

SAS6 AND CW CONCLUSIONS

The current manuscript routinely equates SAS6 levels to cartwheel growth. This is overstated and EM is required to understand whether this is truly impacting the actual cartwheel structure. Loading more sas6 protein doesn't necessarily mean the cartwheel structure changed.

CONNECTION BETWEEN OSCILLATIONS AND MT GROWTH?

Much as above, the manuscript infers MT growth without ever showing it. How does all of this relate to centriole length and growth dynamics.? Page 8 refers to prior work but it seems like this is necessary with EM or MT markers. Having this comparison seems important to the conclusion that MTs do not stop growing when CP110/Cep97 levels reach a threshold level at the distal end.

The following statement is overstated when the data for MT growth are not even presented in this study. "...our findings essentially rule out the possibility that centriole MTs stop growing when a threshold level of CP110/Cep97 accumulates at the centriole distal end."

© 2022. Published by The Company of Biologists under the terms of the Creative Commons Attribution License (https://creativecommons.org/licenses/by/4.0/).

To make such arguments in this study the manuscript would need to include EM and / or MT staining.

ENDOGENOUS UNTAGGED PROTEIN AFFECTING DYNAMICS?

The manuscript shows protein dynamics under conditions of both overexpressed and expression under the endogenous promoter. However, I believe that both of these conditions are also in the presence of untagged protein expression.(?). If so, is it possible that the dynamics represent competition for binding relative to the endogenous, untagged protein? I think this point should at least be discussed.

CP110/Cep97 "INFLUENCED" BY CCO

While I agree that it is likely to be the case that CP110/Cep97 rise and fall at the daughter centriole correlates with CCO, this study does not directly test if CCO changes impact CP110/Cep97 dynamics. Stating that "CP110/Cep97 oscillation is strongly influenced by the activity of the core Cdk/Cyclin cell cycle oscillator (CCO)" is overstated. Is does correlate though.

DISTINCTION FROM PRIOR STUDIES

Dobbelaere 2020 argue that CP110/Cep97 gets to the centriole distal end in late S phase. How could this be considering the data presented in this study? Need discussion of this point. Could Dobbelaere be following the dynamics of the core / basal levels and missed the dynamics that are found in this study? I think a discussion of the Cep97 functions needs to be provided.

MECHANISM OF CROSS TALK

How two apparently spatially separated complexes influence each other should be more mechanistically addressed through either or both experimentation and / or discussion. Obviously the impact of this study would greatly benefit by showing how they are associated and influence each other. CP110 is a phospho target of Plk4. Does this occur in the fly syncytium? Do these interact? What is the timing of the interaction and phosphorylation? Are the changes to SAS6 levels actually the result of Plk4 changes? At this point, these concepts are not tested.

BACKGROUND

In its current form the prior results that 1) Plk4-induced centriole amplification requires CP110 and 2) Plk4 phosphorylates CP110 is important for centriole assembly in some systems is not highlighted in this manuscript as further support for the model of interplay between CP110/Cep97, Plk4 and SAS6.

REPRODUCTION OF DATA

I believe that the data and methods are of high quality and described in such a way that they can be reproduced.

MINOR

ALTERNATIVE MODEL

Because CP110 is a target of Plk4, I wonder if the elevated expression of CP110 sequesters Plk4 away from its cartwheel functions (Ana2/STIL/SAS5 phosphorylation) and this is therefore affecting SAS6 levels?

OVERSTATED CROSSTALK

The text states a "...reveals an unexpected crosstalk between proteins that are usually thought to influence the proximal end of the CW and the distal end of centriole MTs." This is true but there are enough data in the literature to suggest that CP110/Cep97 influence centriole assembly that would indicate that this is not "unexpected".

© 2022. Published by The Company of Biologists under the terms of the Creative Commons Attribution License (https://creativecommons.org/licenses/by/4.0/).

PAGE 11 - SHIFT IN PEAK

I could not find the data clearly showing that there was a shift in "the Plk4 oscillation to later in S-phase". Are the authors referring to the plateau in levels? Please explain further.

WHAT IS "Plk4-NG"?

I assume Neon Green but I don't see the definition.

FIGURE 2

A schematic of the system used for image averaging would help the reader to understand that these "oscillations" represent the mother and daughter centriole together and that each "oscillation" represents one event of the daughter centriole only increasing in CP110/CEP97 levels and then decreasing after peak intensity.

FIGURE 5 and 8

I think these could be supplemental images. I was unable to figure this out but something is wrong with the legend in Figure 8. (A) is referencing items that I cannot find in the figure.

Significance

This study's advance is an expansion of the authors' prior work showing that during the fly nuclear cycles centriole assembly proteins increase and then reduce in what the authors call an oscillation. Here they show that the CP110/Cep97 complex also oscillates and somehow influences the levels of Plk4 and SAS4 that typically reside at the proximal end of the centriole. This is consistent with prior work indicating that, in some systems, CP110/Cep97 influence centriole duplication and assembly.

I believe that with additional experiments to strengthen the conclusions and toned down concluding statements this will be of interest to the centriole, centrosome, and cilia community. My research expertise is also in this community but I am not a Drosophila researcher. I do appreciate the beauty of this system that the authors use.

Reviewer 4

Evidence, reproducibility and clarity

The authors report that CP110 and Cep97 localize near the distal end of centrioles in Drosophila embryos. CP110 and Cep97 tagged with GFP exhibit an oscillatory distribution, with levels on the daughter centriole being maximal in mid S-phase. These oscillations correlate with cell cycle progression. The authors also show that modulating CP110 or Cep97 levels changes the rate at which Sas6-GFP incorporates in the daughter centriole, as well as aspects of the previously reported oscillatory behavior of Plk4.

These results could be of potential interest if the stated conclusions were backed up by more convincing data than that which is provided at present. The issues delineated hereafter must be addressed in full before further consideration of the manuscript.

Major points

1) The oscillatory amplitude of CP110/Cep97 tagged with GFP is much smaller when expression is driven by the endogenous promoters than upon overexpression (see Figure 4), raising the possibility that oscillation might not reflect, or only reflect in part, the behavior of the endogenous proteins. To address this issue, the authors could GFP tag the endogenous loci using CRISPR/Cas9. If this is too demanding, they should at the minimum conduct experiments with the extant lines driven by the endogenous promoters, but in the background of the available CP110 or Cep97 null mutants. Moreover, the authors should stain staged wild-type embryos with antibodies against CP110 and Cep97 to ensure that the mild oscillations reported in Figure 4 do not merely

© 2022. Published by The Company of Biologists under the terms of the Creative Commons Attribution License (https://creativecommons.org/licenses/by/4.0/).

reflect the behavior of the tagged proteins, for example due to the presence of GFP. Related to this point, the authors should considering showing first the data with CP110-GFP GFP-Cep97 driven from the endogenous promoters (current Figure 4), perhaps relegating the results upon overexpression (current Figure 2) to a Supplementary Figure.

2) In repeating the above experiments, the authors must ensure that potential mild oscillations do not simply reflect the fact that centrioles are located at a slightly different distance from the coverslip as a function of cell cycle stage. This could be addressed for example by simultaneously imaging a mother centriole marker such as Asl-mCherry.

Other important points

3) The authors mention (for instance on p. 3) that the inner cartwheel and the surrounding microtubules assemble at opposite ends of the daughter centriole. However, my understanding is that the short centrioles present in the fly embryo have an inner cartwheel that extends throughout the organelle, such that it might be moot to make a distinction between the two ends in this case. Moreover, it is also my understanding that this inner cartwheel is itself surrounded by microtubules, so that microtubule assembly might not be expected to occur strictly at the distal end no matter what.

4) Partially related to the point above, the schematic representations in Figure 1 are somewhat confusing. The schematic in Figure 1A represents CP110/Cep97 strictly at the distal end of the centriole, yet the actual immunofluorescence data on the left suggests that CP110/Cep97 are in fact present very close to Asl-mCherry. This apparent difference must be resolved. Moreover, Figure 1C seems to indicate that all the depicted proteins are present throughout the centriole, which I guess is not what the authors wanted to convey.

5) For the quantification of the data reported in Figure 1, the authors considered only centrioles for which CP110/Cep97 ring eccentricity was less than 1.2, to ensure that only near top views are considered (see p. 23). This is entirely reasonable, but the authors should report the distribution of eccentricities in the data set for the two proteins, and compare them to those of the Sas6-GFP and Sas4-GFP data set, all the more since the latter two were obtained previously with a different microscope modality, potentially complicating thorough comparisons. A slight difference in the fraction of centrioles with a slight tilt could easily skew the data when dealing with such small dimensions.

6) In Figure 3, the authors chose not to report the "Noisy data" observed during mitosis. While it is understandable that the data is noisier at this stage, it must nevertheless be reported, as this may have bearing on assessing oscillations between cycles 12 and 13.

7) The authors should conduct Airy-scan analyses of CP110/Cep97 oscillations driven from the endogenous promoters, to ensure that the variations across the cell cycle reported in Figure 4 reflect changes in the daughter centriole. Moreover, it was not clear why the authors used the Airy-scan for some super-resolution experiments and 3D-SIM for others.

8) Why are solely 1-14 centrioles per embryo considered in the experiments reported in Figure 4 as compared to over 100 per embryo in Figure 2? And how were these centrioles chosen? This needs to be explained, justified and, potentially, rectified.

9) Likewise, why are only the 10 brightest centriole pairs in each embryo retained for the analysis reported in Figure S2? And would the conclusion differ if more centrioles than that were included? Moreover, S phase of cycle 14 is analyzed in Figure S2 for Sas6-GFP, whereas the remainder of the manuscript analyzes CP110/Cep97 during cycles 11 through 13 (with an emphasis on cycle 12). This must be resolved.

10) The Western blots in Figure 4A, 4B, as well as in Figure S1A, should be quantified in the same manner as those in Figure 8C, to achieve a better assessment of the differences in protein levels between conditions.

© 2022. Published by The Company of Biologists under the terms of the Creative Commons Attribution License (https://creativecommons.org/licenses/by/4.0/).

11) The set up for the experiment reported in Figure 8 comes across as a straw man. What one would really like to find out is whether levels of Plk4 at centrioles are modulated by CP110/Cep97 levels, as the authors themselves acknowledge. Since this does not appear to be feasible, the authors set out to test whether cytoplasmic levels of Plk4 differ, finding that this is not the case. Since this experiment does not address what should be tested, it could be reported as a Supplementary Figure, not as the last main figure of the manuscript.

Minor points

- The authors forgot to mention the Tang et al. paper (doi: 10.1038/ncb1889) when referring to Sas-4/CPAP (for instance on p. 4).
- On p. 9, the authors conclude that the "recruitment of CP110/Cep97 to centrioles is regulated by the CCO". Figure 5 shows that the two correlate, not that the latter regulates the former. A related comment holds for the discussion (bottom of p. 13).
- It is not clear why the authors sometimes report SDs (Figure 7) and sometimes SEMs (Figure 3), or fail to report what is being shown (Figure 2). This needs to be clarified.
- The legend of Figure 8A mentions Pie charts and other things that are not featured in the current rendition of the figure.

Significance

These results could be of potential interest if the stated conclusions were backed up by more convincing data than that which is provided at present.

<u>Author response to reviewers' comments</u>

1. General Statements [optional]

We thank the reviewers for their thoughtful and constructive comments and have now revised our manuscript accordingly. We apologise that it has taken so long to send in these revisions, but this is in part because both first authors have now left the lab.

2. Point-by-point description of the revisions

<u>Reviewer #1</u>

This reviewer was generally supportive. They note that it is unfortunate that our data suggests the CP110/Cep97 complex does not play a major part in controlling daughter centriole growth—although we believe that this is an important negative result—but feel that other aspects of our data are interesting. They requested no further experiments, but did comment that it would be interesting to determine when □-tubulin is incorporated into growing centrioles. Unfortunately, we cannot test this as the centrioles in these embryos recruit large amounts of □-tubulin to their PCM, so we cannot specifically assay the small amount of protein in the centriolar fraction.

<u>Reviewer #2</u>

<u>Major Points:</u>

Figure 1: The reviewer notes that Sas-4 and CP110 have antagonistic roles in promoting/repressing centriole growth and asks if Sas-4 is involved in promoting centriole elongation and whether it also oscillates. It is unclear if Sas-4 directly promotes centriole elongation in flies. We have previously shown that centriolar Sas-4 levels do oscillate during S-phase, but with a timing that is distinct from CP110/Cep97 (Novak et al., *Curr. Biol.*, 2014). These observations do not shed much light on the potential antagonistic relationship between CP110/Cep97 and Sas-4, so we do not comment on this here.

Figure S1B: The reviewer requests that we image the centrioles with greater laser intensity to test whether some residual CP110 or Cep97 protein can be recruited in the absence of the other protein. The quantification of this data suggests that some residual CP110 or Cep97 can still be recruited to centrioles in the absence of the other (Graphs, Figure S1B,C), so we do not think it

© 2022. Published by The Company of Biologists under the terms of the Creative Commons Attribution License (https://creativecommons.org/licenses/by/4.0/).

necessary to repeat this experiment at higher laser intensity to further test this point. We now state that the centriolar recruitment of one protein may not be completely dependent of the other (p6, para.2).

Figure 3: The reviewer questions whether the reduction in CP110/Cep97 levels at the mother centriole that we observe during S-phase could be due to photobleaching. This is an interesting point that we now analyse in more detail (p8, para.2). We do not think the decrease in mother centriolar CP110/Cep97 levels is due to photobleaching as our new analysis (which includes more data points during mitosis) strongly suggests that centriolar levels on the mother rise again at the start of the next cycle (New Figure 3C,D).

The reviewer asks whether the CP110/Cep97 oscillations occur at the tip of the growing centriole, and whether we can use super-resolution imaging to address this. A large body of evidence indicates that CP110/Cep97 are highly concentrated at centriole distal tips, and all our experiments suggest that it is this fraction that is oscillating. In Figure 3, for example, we use Airy-scan super-resolution imaging to follow the oscillation on Mother and Daughter centrioles in living embryos. Although the resolution in these experiments is not as high as we can achieve using 3D-SIM in fixed cells, it seems reasonable to assume that the dots of fluorescence we observe oscillating on these centrioles (Fig. 3) are the same fluorescent dots we observe localised at the distal tips of the mother and daughter using 3D-SIM in fixed cells (Fig. 1A).

The reviewer requests additional quantification of the western blots shown in Figure S1 that we use to judge relative expression levels. As we now describe in more detail in the M&M, these ECL blots are very sensitive, but highly non-linear, so we usually estimate relative expression levels by comparing serial dilutions of the different fractions (see, for example, Figure 1B, Franz et al., JCB, 2013). As we now clarify, the key point is not precisely by how much these proteins are over- or under-expressed, but that we observe a similar oscillatory behaviour when they are either over- or under-expressed.

The reviewer points out that our statement that the CP110/Cep97 oscillation is entrained by the Cdk/Cyclin oscillator (CCO) is too strong as it is based only on a correlation. We agree and apologise for this overstatement. To address this, we have now perturbed the CCO by halving the dose of Cyclin B (New Figure 5E—H). This extends S-phase length and we now show that the period of the CP110/Cep97 oscillation is also extended. This suggests that the CCO directly influences the period of the CP110/Cep97 oscillation.

The reviewer notes that our conclusion that the centriole cartwheels are longer or shorter when CP110 or Cep97 are absent or overexpressed, respectively, is based only on Sas-6- GFP fluorescence intensity. They ask if this fluorescence intensity perfectly reflects cartwheel length, and if we can confirm these conclusions using EM. Sas-6 is the main structural component of the cartwheel, so the amount of Sas-6 at the centriole should be proportional to cartwheel length, and we have published two papers that support this conclusion and that use the incorporation of Sas-6 as a proxy to measure cartwheel length (Aydogan et al., *JCB*, 2018; Aydogan et al., *Cell*, 2020). Importantly, our previous EM studies support our conclusions about the relationship between cartwheel length and CP110/Cep97 levels: the centrioles in wing-disc cells are slightly longer in the absence of CP110 and slightly shorter when CP110 is overexpressed (Franz et al., *JCB*, 2013). The new findings reported here provide a potential explanation for this EM data, which was puzzling at the time.

Minor Points:

Figure 1C: The reviewer noted that our schematic illustrations in this Figure could be misleading. We agree and have now redrawn them.

<u>Reviewer #3</u>

<u>Major points:</u>

The reviewer requested that we clarify our use of the term oscillation, pointing out that oscillations are repetitive variations in levels/activity over time, whereas the “oscillations” we

© 2022. Published by The Company of Biologists under the terms of the Creative Commons Attribution License (https://creativecommons.org/licenses/by/4.0/).

describe here occur during each round of centriole assembly. This is a fair point, and one that is often debated in the oscillation field, with many believing that too many biological processes are termed "oscillations", when they are not truly driven by the passage of time. To avoid any ambiguity, we now no longer describe the behaviour of CP110/Cep97 as an oscillation (although, for ease of discussion, we still use the term in this letter).

The reviewer thought that the data we show in Figure 1 was not relevant as we largely analyse centrioles in living embryos whereas the data in Figure 1 is derived from fixed wing-disc cells—and similar fixed-cell data has been shown in previous studies. The reviewer suggests we use super-resolution methods to analyse Cp110/Cep97 dynamics in the syncytial embryo, and show this relative to Sas-6 and Plk4. They ask if Plk4 and CP110/Cep97 colocalise at any time. While CP110/Cep97 localisation has been analysed by super-resolution microscopy previously (e.g. Yang et al., *Nat. Comm.*, 2018; LeGuennec et al., *Sci. Adv.*, 2020), CP110/Cep97 was a minor part of these studies and our data is the first to show that this complex sits as a ring on top of the centriole MTs in fly centrioles (that lack the complex distal and sub-distal appendages present in the previously analysed systems). As this localisation is important in thinking about how CP110/Cep97 might influence centriole MT growth, we would like to include it. We cannot show this detail in living embryos as the movement of the centrioles reduces resolution and we cannot observe the ring structure.

Although we do use Airy-scan super-resolution microscopy to study CP110/Cep97 dynamics in living embryos (Figure 3), we cannot do this in two colours (to compare these dynamics to Sas-6 or Plk4 dynamics) as red-fluorescent proteins bleach too quickly. We now show the relative dynamics of CP110/Cep97 and Plk4 recruitment using standard resolution microscopy (New Figure S2). While it is well established that Plk4 and CP110/Cep97 are concentrated at opposite ends of centrioles, they are all recruited to the nascent site of daughter centriole assembly, effectively "colocalising" at this timepoint. This could provide an opportunity for the crosstalk we observe here, and we now mention this possibility (p17, para.1).

The Reviewer questioned whether the loading of Sas-6-GFP onto centrioles can be used as a proxy for cartwheel length, pointing out that Sas-6 could load into centrioles in a way that does not change the cartwheel structure, and that EM is required to test this. As described in our response to Reviewer #2, Sas-6 is the main structural component of the cartwheel, and we have published two papers that use the incorporation of Sas-6 into the cartwheel as a proxy to measure cartwheel length (Aydogan et al., *JCB*, 2018; Aydogan et al., *Cell*, 2020). While we cannot exclude that Sas-6 might also associate with the cartwheel in a way that does not involve its incorporation into the cartwheel, it is not clear how EM might address this question. Moreover, even if such a fraction existed, it should not affect our conclusions—as long as Sas-6 is binding to the cartwheel in some way, then the amount bound should remain proportional to the length of the cartwheel. Perhaps the reviewer is suggesting that we perform an EM time course of cartwheel growth to back up our conclusions from the Sas-6 incorporation assay? If so, we think this impractical. The changes in cartwheel length shown in Figure 6 are revealed from analysing several thousand images of centrioles compared at precise relative time points. Such an analysis cannot be done in fixed embryos by EM.

Similar to the point above, the reviewer notes that we use the length of the cartwheel to infer centriole MT length, but we never directly measure MT length. They suggest we perform either an EM analysis or use MT markers to directly measure the kinetics of centriole MT growth. In flies (and many other organisms), the centriole MTs grow to the same length as the centriole cartwheel (Gonzalez, *JCS*, 1998), so we can be confident that the final length of the cartwheel reflects the final length of the centriole MTs. Moreover, we previously measured the distance between the mother centriole and the GFP-Cep97 cap that sits at the distal tip of the centriole MTs as a proxy for centriole MT length, and found that the inferred kinetics of MT growth were similar to the kinetics of cartwheel growth (inferred from Sas-6 incorporation) (Aydogan et al., 2018). This manual analysis was very time consuming, and we have tried to implement computational analysis methods, but so far without success. For similar reasons to those described in the point above, it is not feasible to accurately measure centriole MT growth kinetics by EM (nobody has been able to do this). Moreover, the centrosomes in these embryos are associated with too much tubulin and the centriole MTs are not yet modified (e.g. by acetylation) as the cycles are so fast—so we cannot directly stain the centriole MTs in fixed embryos. We have now

© 2022. Published by The Company of Biologists under the terms of the Creative Commons Attribution License (https://creativecommons.org/licenses/by/4.0/).

toned down our conclusions about MT length throughout the paper, and we make it clear that we cannot directly measure this.

All of the experiments shown here are performed in the presence of endogenous untagged proteins, and the reviewer wonders if recruitment dynamics might be influenced by competition for binding from the endogenous protein. We have compared the behaviour of many centriole and centrosome proteins in the presence and absence of the untagged WT protein. In all cases, less tagged-protein binds to centrioles/centrosomes in the presence of untagged protein, presumably due to competition. Apart from this, however, we usually observe no real difference in overall dynamics and in Reviewer Figure 1 (see below) we show that CP110- GFP and GFP-Cep97 both oscillate even in the absence of any endogenous protein. As we feel this result is not very surprising, we do not show it in the manuscript.

The reviewer correctly noted that our data was not strong enough to conclude that the CP110/Cep97 oscillation is influenced by the CCO. This was also raised by Reviewer #2 and, as described above (p2, para.3 above), we have now performed additional experiments to more directly demonstrate this point (new Figure 5G—H).

The reviewer requests more discussion of why our conclusion that CP110/Cep97 levels oscillate on the growing daughter centrioles during S-phase is different to that reached by Dobbelaere et al, (*Curr. Biol.*, 2020), who conclude that Cep97-GFP only starts to incorporate into the new daughter centrioles late in S-phase when the daughters are fully grown. We have discussed this discrepancy with these authors and they kindly shared their reagents with us (so our endogenous Cep97-GFP oscillation data comes from the same line they used in their experiments), but we have not come to a clear conclusion on this point. We have shown robust oscillations for CP110 and Cep97 by quantifying many hundreds of centrioles using multiple transgenes (both over- and under-expressed) in multiple backgrounds. Cep97 dynamics were a very minor part of the Dobbelaere et al., study, and they analysed a much smaller number of centrioles. We now briefly mention this discrepancy (p9, para.1), but do not discuss it in detail as we have no definitive explanation for it.

The reviewer requests more experiments or more discussion to address the mechanism(s) of crosstalk between CP110/Cep97 and Plk4, and they suggest several avenues for further investigations. These are excellent ideas, and we are working hard on these approaches. These are all long-term experiments, however, and we feel it is important that the field be made aware of these surprising findings as soon as possible, as others may be better-placed to provide mechanistic insight into how this system ultimately works. We now briefly mention some of the future directions the reviewer highlights in the Discussion.

The reviewer thought we should highlight the previous publications showing that Plk4-induced centriole amplification requires CP110 and that Plk4 can phosphorylate CP110. These studies (Kleylein-Sohn et al, *Dev. Cell*, 2007; Lee et al., *Cell Cycle*, 2017) were mentioned, but we now discuss them more prominently (p17, para.2).

Minor Points:

The reviewer raised a number of minor concerns that we have now addressed: (1) We discuss the model the reviewer suggests; (2) we no longer state that the crosstalk between CP110/Cep97 and Plk4 is unexpected; (3) We have clarified our description of the shift in timing of the peak levels of CP110/Cep97, which we no longer refer to as an oscillation; (4) We define mNG as monomeric Neon Green; (5) We have changed our schematics in Figure 1 as suggested by the reviewer; (6) We have corrected the mistake in the legend to Figure 8.

Reviewer #4

Major points:

1. The reviewer noted that the amplitude of the CP110/Cep97 oscillations depended on protein expression levels, so the oscillations might not reflect the behaviour of the endogenous proteins. They requested that we either repeat our experiments with CRISPR

© 2022. Published by The Company of Biologists under the terms of the Creative Commons Attribution License (https://creativecommons.org/licenses/by/4.0/).

knock-in alleles, or conduct experiments with the lines driven by the endogenous promotors but in their respective mutant backgrounds. We have not generated CRISPR knock-ins for CP110/Cep97, but have done so for many other centriole/centrosome proteins (>8) and found that most such lines are expressed at higher or lower levels than the endogenous allele (and sometimes very significantly so). This is also true for our standard transgenic lines, where genes are expressed from their endogenous promoters, but are randomly integrated into the genome. The blots in Figure 4 show that CP110-GFP and GFP-Cep97 expressed from a ubiquitin (u) promoter or from their endogenous promoters (e) are expressed at ~2-5X higher or ~2-5X lower levels than the endogenous proteins, respectively. As we observe CP110/Cep97 oscillations in all cases, it seems unnecessary to generate new CRISPR knock-ins (that are also likely to be somewhat over- or under-expressed) to show this again. As the reviewer asks, we show that Cep97-GFP and CP110-GFP still oscillate in in the absence of the endogenous proteins (Reviewer Figure 1). As this does not seem a surprising result, we do not show this in the main manuscript.

In the same point the reviewer requests that we use antibody staining in fixed embryos to show that the untagged proteins also oscillate. Analysing protein dynamics is much harder in fixed embryos, as the levels of fluorescent staining are more variable and we can only approximately infer relative timing, rather than precisely measuring it (as we can in living embryos). Moreover, as both proteins in the CP110/Cep97 complex exhibit a very similar oscillatory behaviour when tagged with either GFP or RFP (e.g. Figure 2C), and this behaviour is distinct to that observed with several other GFP- or RFP-tagged centriole proteins (e.g. Novak et al., *Curr. Biol.*, 2014; Conduit et al., *eLife*, 2015; Aydogan et al., *JCB*, 2018; Aydogan et al., *Cell*, 2020) it seems very unlikely that this behaviour is induced by the GFP (or RFP) tag.

The reviewer also suggests that we show the data with the endogenous promoter before we show the data with the ubiquitin promoter. As we now explain better (and show in Figure 4), this seems unnecessary as the proteins expressed from the ubiquitin promotor are probably actually expressed at levels that are more similar to the endogenous protein.

2. The reviewer questions whether the oscillations we observe might be due to the centrioles simply moving up and down in the embryo during the cell cycle, and they suggest we monitor Asl behaviour to rule this out. We have previously shown that Asl-GFP levels do not oscillate; they remain constant throughout the cell cycle on old-mother centrioles, and grow approximately linearly throughout S-phase on new-mother centrioles (see Figure 1D in Novak et al., *Curr. Biol.*, 2014).

3. We were not sure we understood this point properly, so we copy the reviewers comment in full here: ***The authors mention (for instance on p. 3) that the inner cartwheel and the surrounding microtubules assemble at opposite ends of the daughter centriole. However, my understanding is that the short centrioles present in the fly embryo have an inner cartwheel that extends throughout the organelle, such that it might be moot to make a distinction between the two ends in this case. Moreover, it is also my understanding that this inner cartwheel is itself surrounded by microtubules, so that microtubule assembly might not be expected to occur strictly at the distal end no matter what.*** The reviewer is correct that *Drosophila* centrioles are short (~150nm) and that the cartwheel extends throughout the centriole. We think the reviewer is suggesting that it may not be relevant therefore whether the cartwheel and centriole MTs grow from opposite ends—as the activities that govern their growth may not be spatially separated? However, because cartwheels grow preferentially from the proximal-end (Aydogan et al., JCB 2018) while centriole MTs are assumed to grow preferentially from the distal (plus) end, there is an intrinsic problem in ensuring they grow to the same size—no matter how short or long the centrioles are. The reviewer is correct that one possible solution to this problem is that the centriole MTs actually grow from their minus ends, but this is not widely accepted (or even proposed). We have tried to explain this issue more clearly throughout the revised manuscript.

4. The reviewer points out that the schematic illustrations in Figure 1A and 1C are inaccurate and unhelpful. We agree and have now redrawn these.

5. The reviewer asks that we provide information about the eccentricities of the centrioles in the different datasets used to calculate the protein distributions shown in Figure 1,

© 2022. Published by The Company of Biologists under the terms of the Creative Commons Attribution License (https://creativecommons.org/licenses/by/4.0/).

particularly as the data for Sas-4-GFP and Sas-6-GFP were obtained previously using a different microscope modality, making comparisons complicated. The point that comparing distance measurements across different datasets is difficult is an important one, and we now state that such comparisons should be treated with caution. However, we have not provided information on the distribution of centriole eccentricities in the different experiments as it wasn't clear to us how this information could be used to make such comparisons more accurate (presumably the reviewer is suggesting we could apply a correction factor to each dataset?). The very tight overlap in the positioning of CP110/Cep97 fusions (Figure 1C) strongly suggests that any difference in the average centriole eccentricities of the different populations of centrioles analysed, which are already tightly selected for their *en-face* orientation (i.e. eccentricity <1.2), are unlikely to be a significant source of error in this experiment.

6. **The reviewer requested that we show the "noisy data" we obtained during mitosis that we excluded from our analysis in Figure 3.** As we now explain in more detail (p8, para.2), there are two reasons why the data for mitosis in this experiment is "noisy": (1) The protein levels on the centrioles are low in mitosis and the centrioles are more mobile, so they are hard to track; (2) The Asl-mCherry marker used to identify the mother centriole starts to incorporate into the daughter (now new mother) centriole during mitosis, making it difficult to unambiguously distinguish mothers and daughters. As a result, we cannot track and assign mother/daughter identity to very many centrioles during mitosis—although we now include some extra data-points during mitosis for the centrioles where we could do this (revised Figure 3C,D). Importantly, it is clear that this "noisy" data hides no surprises: one can see (Figure 3C,D) that the signal on the centrioles is simply low during mitosis and then starts to rise again as the embryos enter the next cycle. This is confirmed in the normal resolution data (Figure 2B,C; Movies S1 and S2) where we can track many more centrioles due to the wider field of view and because we do not have to discard centrioles in mitosis that we cannot unambiguously assign as mothers or daughters.

7. **The reviewer requests that we conduct a super-resolution Airy-scan analysis of CP110/Cep97 driven from their endogenous promoters (eCP110 or eCep97) to ensure that the oscillations we see with these lines (shown in Figure 4C,D) are also occurring at the daughter centriole—as we already show for the oscillations observed with the uCP110 and uCep97 lines (shown in Figure 4C,D, and analysed at super-resolution on the Airy-scan in Figure 3).** This is technically very challenging as super-resolution techniques require a lot of light and the centriole signal in the eCP110/Cep97 embryos is very dim compared to uCP110/Cep97 embryos (Figure 4C,D). We have managed to do this for eCep97-GFP and confirmed that—even in these embryos that express Cep97-GFP at much lower levels than the endogenous protein (Figure 4A)—the "oscillation" is primarily on the daughter (Reviewer Figure 2). As this data is very noisy, and as the ubiquitin uCP110/Cep97 lines express these fusions at levels that are closer to endogenous levels (Figure 4A,B), we do not show this data in the main text.

The reviewer also asks for clarification as to why we use the Airy-scan for some experiments and 3D-SIM for others. As we now explain (p8, para.1), 3D-SIM has better resolution than the Airy-scan, but it takes more time and requires more light—so we cannot use it to follow these proteins in living embryos. Thus, for tracking CP110/Cep97 throughout S-phase in living embryos we had to use the Airy-scan.

8. **The reviewer questions why in some experiments we analyse the behaviour of 100s of centrioles, whereas in others the numbers are much smaller (1-14 in Figure 3—*note*, the reviewer quoted this number as coming from Figure 4, but it actually comes from Figure 3, so we have assumed they mean Figure 3).** We apologise for not explaining this properly. The super-resolution experiments in Figure 3 are performed on a Zeiss Airy-scan system, which has a much smaller field of view than the conventional systems we use in other experiments. Thus, we inherently analyse a much smaller number of centrioles in these experiments. In addition, as explained in point 6 above, in these experiments we need to analyse mother and daughter centrioles independently, and in many cases we cannot unambiguously make this assignment, so these centrioles have to be excluded from our analysis.

9. **The reviewer questions why we selected the 10 brightest centrioles for the analysis shown in Figure S1B,C (*note*, the reviewer states Figure S2 here, but it is the data shown in Figure S1B,C that is selected from the 10 brightest centrioles, so we assume this is the**

© 2022. Published by The Company of Biologists under the terms of the Creative Commons Attribution License (https://creativecommons.org/licenses/by/4.0/).

relevant Figure). We apologise for not explaining this properly. In these mutant embryos very little CP110-GFP localises to centrioles in the absence of Cep97, and *vice versa*, so we cannot track centrioles using our usual pipeline and instead have to select centrioles using the Asl-mCherry signal. As the difference between the WT and mutant embryos is so striking, we simply selected the brightest 10 centrioles (based on Asl-mCherry levels) in both the WT and mutant embryos for quantification. We could select more centrioles, or select centrioles based on different criteria, but our main conclusion—that the centriolar localisation of one protein is largely dependent on the other—would not change.

The reviewer also questioned why we performed the analysis shown in Figure S2 (new Figure S3) during S-phase of nuclear cycle 14, when the rest of the manuscript focuses on nuclear cycles 11-13. We apologise for not explaining this properly. In cycles 11-13 centriolar CP110/Cep97 levels rise and fall during S-phase, whereas both proteins reach a sustained plateau during the extended S-phase (~1hr) of nuclear cycle 14—making it easier to analyse CP110/Cep97 levels in embryos when their centriole levels are maximal. We now explain this.

10. The reviewer requests that we quantify the western blots shown in Figure 4 in the same way we do in figure 8. To do this we would need to perform multiple repeats of these blots and we did not perform these because the blots shown in Figure 4 largely recapitulate already published data (Franz et al., *JCB*, 2013; Dobbelaere et al., *Curr. Biol.*, 2020). Moreover, as described in our response to Reviewer #2, these ECL blots are very sensitive, but highly non-linear, so we always compare multiple serial dilutions of the different extracts to try to estimate relative levels of protein expression. We now explain this in the M&M.

11. The reviewer suggests the data shown in Figure 8 is a "straw man": we really want to test whether modulating CP110/Cep97 levels modulates *centriolar* Plk4 levels, but instead we test how they modulate *cytoplasmic* Plk4 levels. The language here is harsh, as it suggests that our intention was to mislead readers into thinking that we have addressed a relevant question by addressing a different, irrelevant, one. We apologise if we have missed something, but we believe we do perform exactly the experiment that the reviewer thinks we should be doing— quantifying how *centriolar* Plk4 levels change when we modulate the levels of CP110 or Cep97 (Figure 7). It is clear from this data that modulating the levels of CP110/Cep97 does indeed modulate the centriolar levels of Plk4. In Figure 8 we seek to address whether this change in centriolar Plk4 levels occurs because global Plk4 levels in the embryo are affected—a very reasonable hypothesis, which this experiment addresses quite convincingly (although negatively).

Minor Points:
The reviewer highlights a small number of mistakes and omissions, all of which have been corrected.

Finally, we would like to thank the reviewers again for their detailed comments and suggestions. We hope that you and they will agree that the changes we have made in response to these comments have substantially improved that manuscript and that it is suitable for publication in The Journal of Cell Science.

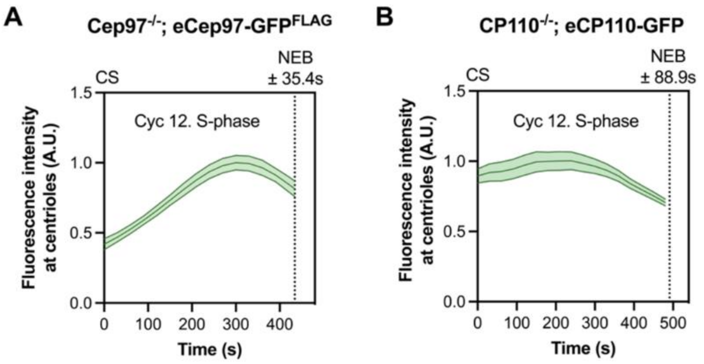

Reviewer Figure 1. CP110/Cep97 dynamics remain cyclical even when Cep97-GFP and CP110-GFP are expressed from their endogenous promotors in the absence of any endogenous

© 2022. Published by The Company of Biologists under the terms of the Creative Commons Attribution License (https://creativecommons.org/licenses/by/4.0/).

protein. Graphs show how the levels (Mean±SEM) of centriolar CP110/Cep97- GFP change during nuclear cycle 12 in (A) *Cep97*$^{-/-}$ embryos expressing eCep97-GFP or (B) *CP110*$^{-/-}$ embryos expressing eCP110-GFP. CS=Centrosome Separation, NEB=Nuclear Envelope Breakdown. N≥11 embryos per group, average of n≥15 centrioles per embryo.

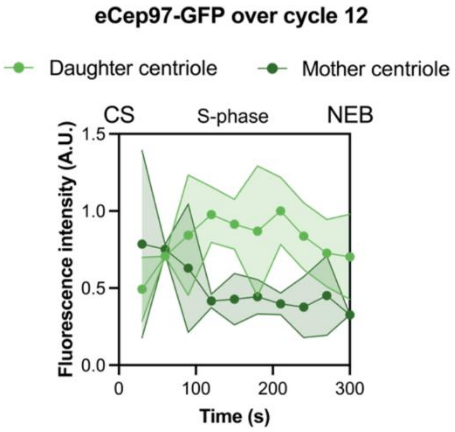

Reviewer Figure 2. The cyclical recruitment of Cep97-GFP expressed from its endogenous promoter occurs largely at the growing daughter centriole. The graph quantifies the fluorescence intensity (Mean±SD) acquired using Airy-scan microscopy of eCep97-GFP on mother (dark green) and daughter (light green) centrioles in individual embryos over Cycle 12. CS = Centrosome Separation, NEB = Nuclear Envelope Breakdown. Data was averaged from 3 embryos as the number of centriole pairs that could be measured was relatively low (total of 2-8 daughter and mother centrioles per time point; in part due to the much dimmer signal of eCep97-GFP in comparison to uGFP-Cep97).

Original submission

First decision letter

MS ID#: JOCES/2022/260015

MS TITLE: Centriole distal-end proteins CP110 and Cep97 influence cartwheel growth at the proximal-end of centrioles

AUTHORS: Mustafa G. G Aydogan, Laura E Hankins, Thomas L. L Steinacker, Mohammad Mofatteh, Saroj Saurya, alan wainman, Siu-Shing Wong, Xin Lu, Felix Y Zhou, and Jordan W Raff
ARTICLE TYPE: Research Article

Thank you for submitting your revised work to JCS following previous review, we have now reached a decision on the above manuscript.

To see the reviewers' reports and a copy of this decision letter, please go to: https://submit-jcs.biologists.org and click on the 'Manuscripts with Decisions' queue in the Author Area. (Corresponding author only has access to reviews.)

As you will see, the reviewers gave favourable reports but raised some critical points that will require amendments to your manuscript. I hope that you will be able to carry these out because I would like to be able to accept your paper, depending on further comments from reviewers. I do not consider that the comments require further experiments (despite the suggestion of one reviewer to use a ubiquitin promoter). Clearly any data you could provide relating to endogenous proteins would support the conclusions. There are some points raised that would require further analysis of the data and amendments to the presentation and I broadly agree with those points and support their suggestions.

© 2022. Published by The Company of Biologists under the terms of the Creative Commons Attribution License (https://creativecommons.org/licenses/by/4.0/).

Please ensure that you clearly highlight all changes made in the revised manuscript. Please avoid using 'Tracked changes' in Word files as these are lost in PDF conversion.

I should be grateful if you would also provide a point-by-point response detailing how you have dealt with the points raised by the reviewers in the 'Response to Reviewers' box. Please attend to all of the reviewers' comments. If you do not agree with any of their criticisms or suggestions please explain clearly why this is so.

Reviewer 1

Advance summary and potential significance to field

I have reviewed this manuscript upon request of the Review Commons. I submitted the following critics at that time. The authors sufficiently responded to me the reviewer #1. Therefore, I believe that the manuscript is ready to be published in JCS.

<Critics of the reviewer #1>

This manuscript is a continuation of the previous articles of the authors (Aydogan et al., JCB 217:1233, 2018; Aydogan et al., Cell 181:1566 2020). They reported that Plk4 initiates and times the growth of the cartwheel at the proximal end during early divisions of the Drosophila embryos. In this manuscript, they investigated roles of the CP110/Cep97 complex in the centriole growth control at the distal end of the centriole. The daughter centriole levels of the CP110/Cep97 complex oscillate in S phase in a similar manner to those of Plk4. The CP110/Cep97 oscillation is entrained by the core Cdk/Cyclin cell cycle oscillator but not by Plk4. Rather, the centriolar levels of Plk4 increased in the CP110 and Cep97 deletion embryos. The experiments seem to be carefully carried out, data are nicely presented, and manuscript is clearly written.

I agree with their interpretation that the CP110/Cep97 oscillation does not appear to play a major part in determining the period of daughter centriole growth during early divisions of the Drosophila embryos. The CP110/Cep97 complex seems to have a limited role in the centriole length control. The CP110/Cep97 complex may be important to prevent centrioles from over-elongating after the initial growth of centrioles.

As suggested in the manuscript, phosphorylation may be a regulatory mechanism for CP110 behaviors at the centrioles. It was previously reported that CP110 is a substrate of the cell cycle kinases, such as Cdk2 (Chen et al., Dev Cell 3:339, 2002) and Plk4 (Lee et al., Cell Cycle 16:1225, 2017). Phosphorylation may be required for recruitment or removal of CP110 at the centrioles. Nonetheless, it is hard to interpret the functional significance of the S phase oscillation of the CP110/Cep97 complex with the data in the manuscript.

It is unfortunate to conclude that the CP110/Cep97 complex may not be a major player for controlling the centriole growth. However, the manuscript includes other interesting observations. For example, they presented data supporting that the SAS6 protein is added at the proximal side of the centrioles, which is opposite to the microtubule growth. Microtubules in the daughter centrioles may assemble at the minus end rather than the plus end. It would be interesting to determine when γ-tubulins are recruited to the growing centrioles.

Comments for the author

I have reviewed this manuscript upon request of the Review Commons. I submitted the following critics at that time. The authors sufficiently responded to me, the reviewer #1. Therefore, I believe that the manuscript is ready to be published in JCS.

<Critics of the reviewer #1>

This manuscript is a continuation of the previous articles of the authors (Aydogan et al., JCB 217:1233, 2018; Aydogan et al., Cell 181:1566, 2020). They reported that Plk4 initiates and

© 2022. Published by The Company of Biologists under the terms of the Creative Commons Attribution License (https://creativecommons.org/licenses/by/4.0/).

times the growth of the cartwheel at the proximal end during early divisions of the *Drosophila* embryos. In this manuscript, they investigated roles of the CP110/Cep97 complex in the centriole growth control at the distal end of the centriole. The daughter centriole levels of the CP110/Cep97 complex oscillate in S phase in a similar manner to those of Plk4. The CP110/Cep97 oscillation is entrained by the core Cdk/Cyclin cell cycle oscillator but not by Plk4. Rather, the centriolar levels of Plk4 increased in the CP110 and Cep97 deletion embryos. The experiments seem to be carefully carried out, data are nicely presented, and manuscript is clearly written.

I agree with their interpretation that the CP110/Cep97 oscillation does not appear to play a major part in determining the period of daughter centriole growth during early divisions of the *Drosophila* embryos. The CP110/Cep97 complex seems to have a limited role in the centriole length control. The CP110/Cep97 complex may be important to prevent centrioles from over-elongating after the initial growth of centrioles.

As suggested in the manuscript, phosphorylation may be a regulatory mechanism for CP110 behaviors at the centrioles. It was previously reported that CP110 is a substrate of the cell cycle kinases, such as Cdk2 (Chen et al., Dev Cell 3:339, 2002) and Plk4 (Lee et al., Cell Cycle 16:1225, 2017). Phosphorylation may be required for recruitment or removal of CP110 at the centrioles. Nonetheless, it is hard to interpret the functional significance of the S phase oscillation of the CP110/Cep97 complex with the data in the manuscript.

It is unfortunate to conclude that the CP110/Cep97 complex may not be a major player for controlling the centriole growth. However, the manuscript includes other interesting observations. For example, they presented data supporting that the SAS6 protein is added at the proximal side of the centrioles, which is opposite to the microtubule growth. Microtubules in the daughter centrioles may assemble at the minus end rather than the plus end. It would be interesting to determine when γ-tubulins are recruited to the growing centrioles.

Reviewer 2

Advance summary and potential significance to field

See original review.

Comments for the author

The authors conducted further experiments and analyses to address the comments raised by the four reviewers of the initial submission. The manuscript is clearly improved as a result. However, three important points remain to be clarified in my opinion before publication in JCS could be endorsed, as detailed hereafter.
The numbering of these points refers to that of my initial review.

Major points:
1) Just like reviewer #3, I wondered whether the oscillations of CP110-GFP and GFP-Cep97 expressed from the ubiquitin promoter might not reflect, or reflect only in part, the behavior of the endogenous proteins. The authors argue that the suggested CRISPR knock-ins might not be informative. Instead, they tested the behavior of the tagged proteins driven by their endogenous promoters in the background of CP110 or Cep97 null mutants, as suggested also. The authors report this data in Reviewer Figure 1 and conclude that the proteins still oscillate in such conditions. An examination of this figure suggests slightly otherwise, at the least for CP110, the dynamics of which appears quite different from that reported in Figure 4C. The authors should include this new piece of data in their manuscript, and consider the impact of this apparent oscillation dampening on their interpretation. Perhaps conducting an analogous experiment with the constructs driven by the ubiquitin promoter could shed further light on this question. Furthermore, as mentioned in my initial review, it would be best to corroborate the conclusions drawn from examining the tagged proteins by analyzing the endogenous proteins. There should be no problem to stage fixed embryos using well established markers of cell cycle progression. Such experiments would go a long way towards solidifying these intriguing findings.

© 2022. Published by The Company of Biologists under the terms of the Creative Commons Attribution License (https://creativecommons.org/licenses/by/4.0/).

2) Perhaps my initial comment was not sufficiently clear here: it is because Asl-mCherry levels do not oscillate that monitoring it, for instance in the experiment reported in Figure 3A and 3B, would ensure that potential mild oscillations of CP110 and Cep97 do not simply reflect the fact that centrioles are located at a slightly different distance from the coverslip as a function of cell cycle stage. It appears that the authors should have the data to conduct and report this analysis.
10) Just like Reviewer #1, I requested that the Western blots in Figure 4A, 4B, as well as in Figure S1A, be quantified like those in Figure 8C, to better assess differences in protein levels between conditions. The authors responded that because ECL blots are non-linear, they always compare multiple serial dilutions to estimate relative protein expression levels, something they now mention in the Materials and Methods section. This response prompts two comments. Firstly, if ECL blots cannot be used for quantifications, why are the authors reporting such quantifications in Figure 8C? Secondly, the authors should show in a Supplementary Figure panel the multiple serial dilutions they refer to in the Materials and Methods section, as quantifications of CP110 and Cep97 levels in the different genotypes is a critical aspect of their analysis.

Other points:
- The out of phase behavior of RFP-Cep97 and Plk4-GFP does not appear to be so clear-cut judging from Figure S2. The authors should also report the average of these 5 embryos, just like they do in Figure 2C for CP110-GFP and GFP-CEP97, and potentially tamper their conclusion accordingly.
- The impact of halving the dose of CycB on the position of the peak centre appears marginal (Fig. 5G and 5H), albeit statistically significant. The authors might consider adjusting the wording on page 11 accordingly.

Reviewer 3

Advance summary and potential significance to field

This study uses nuclear cycles 11-13 of Drosophila embryos to show the dynamics of the distal centriole localizing CP110/Cep97 complex during the predicted time of MT assembly during new centriole assembly. Continuing from prior work from this group, the authors find that the increase and decrease in CP110/Cep97 at new centrioles correlates with the timing of Cdk/Cyclin oscillations (CCO). The authors find that increased or decreased levels of CP110/Cep97 changes the dynamics of SAS6 and Plk4 levels. This work suggests that there is crosstalk between the distal localizing CP110/Cep97 complex and the proximal localizing Plk4 and SAS6 proteins required for early centriole assembly.

Comments for the author

This revised manuscript from Aydogan et al addresses most of the comments that I had as a reviewer in the Review Commons. I think that the revision is appropriate for publication in Journal of Cell Science. I do have a couple of comments below.
Figure 1 localization of CP110/Cep97 to the distal end is confusing to me. I don't understand why a radial length measurement of the centriole rotational distribution of these proteins would reveal the localization to the distal end of the proximal-distal centriole axis. I think I am missing something here and wonder if the authors can please clarify this in the figure.
I would consider discussing the caveat that using fluorescence levels of resident proteins of a structure (centriole or CW) as a proxy for defining centriole and CW length may be misleading. I understand the authors' point that this is a difficult EM experiment and that the beautiful dynamics shown could not be achieved here without these live imaging experiments but the potential for misinterpreting (overstating) length changes based on levels of protein at a site exists. My suggestion would be to simply make this point in the results or in the discussion.

© 2022. Published by The Company of Biologists under the terms of the Creative Commons Attribution License (https://creativecommons.org/licenses/by/4.0/).

First revision

Author response to reviewers' comments

Thank you for sending us the Reviewer's comments on our revised manuscript, and sorry that it has taken so long to get back to you. As I mentioned previously, both first authors left the lab some time ago so we faced some logistical challenges. Nevertheless, we are very pleased that the Reviewer's thought the manuscript was significantly improved, and we have addressed their remaining concerns as described below.

Reviewer #1
This reviewer thought the manuscript was now suitable for publication in JCS.

Reviewer #2
This reviewer still had concerns on three **Major Points**, numbered in relation to their first round of comments:

1. The reviewer remains concerned that the oscillations we report here with fluorescently tagged CP110- and Cep97-fusion proteins do not reflect the behaviour of the endogenous proteins. This concern was heightened because the kinetics of the eCP110-GFP oscillation we report in the *CP110*$^{-/-}$ mutant background—shown in *Reviewer Figure [RF] 1*—was not convincing. To do this experiment we had to send these stocks to UCSF (where the original experimentalist, Mustafa Aydogan, is now located) and analyse embryos on a different microscope setup. Frustratingly, although the Cep97 embryos were healthy and the Cep97- GFP oscillation was clear, the CP110 line embryos were very sick and the fluorescence very dim (for unknown reasons, as embryos completely lacking CP110 are not normally sick, and embryos rescued by other CP110-GFP constructs are healthy). Thus, the CP110 data is of unusually poor quality—although we believe it is still possible to discern an oscillation that is consistent with our observations in many other genetic backgrounds that fluorescently tagged-CP110 centriolar levels oscillate (e.g. Figure 2B and 2C; Figure 3A,C; Figure 4C; Figure 5E; Movie S1). We feel it is not necessary to show this data (as the Reviewer requests) because it was collected on a different microscope system and it is of poor quality compared to the other data we show.

Moreover, this experiment does not address the Reviewer's main concern that the untagged proteins do not oscillate. Instead, it only shows that tagged-versions of CP110 and Cep97 oscillate (something we feel we have demonstrated convincingly already, even though the CP110 data in RF#1 is poor). The Reviewer then suggests, again, that it is trivial to show that the endogenous proteins oscillate in fixed embryos. Again, we can assure the reviewer that this is not the case. In fixed embryos there are numerous potential confounding factors (e.g. variation in protein levels in each embryo, how well the embryos are fixed, how well the antibodies penetrate, whether the embryo was cycling slowly or quickly) that make it much harder to demonstrate subtle oscillatory behaviour when compared to an analysis in living embryos—where one can directly measure how fluorescence levels change subtly over time at the same centrioles in the same embryo.

To summarise our response to this point. We believe our data convincingly shows that levels of the CP110/Cep97 complex oscillate at centrioles if either protein in the complex is tagged with GFP or RFP and is expressed at levels that are either slightly higher or slightly lower than the endogenous proteins. As we do not observe a similar oscillatory behaviour with multiple other fluorescently tagged centriole proteins, and as the fluorescently-tagged versions of both CP110 and Cep97 fully rescue their respective mutant phenotypes, we think it unnecessary to undertake the huge amount of work required to test whether the endogenous (untagged) proteins oscillate. We now prominently mention this caveat (p16, end of para.1), and hope that readers can make up their own mind as to how likely it is that this oscillatory behaviour is an artefact induced by the GFP- or RFP-tagging of both proteins.

2. The reviewer was initially concerned that the mild oscillations we observed in the centriole levels of CP110/Cep97 may reflect the centrioles moving closer and then further away from the coverslip during the nuclear cycles. We argued that this was very unlikely, as we have monitored the incorporation of several other centriole proteins, such as Asl (Novak et al., 2014) Sas-6 (Aydogan et al, 2018) and Plk4 (Aydogan et al., 2020), and these either don't oscillate, or

© 2022. Published by The Company of Biologists under the terms of the Creative Commons Attribution License (https://creativecommons.org/licenses/by/4.0/).

oscillate with a different phase to the CP110/Cep97 oscillation (see, for example, Figure S2). The Reviewer has now clarified that they already appreciated that Asl levels don't oscillate as CP110/Cep97 do, but they wanted us to show this again as a control for the CP110/Cep97 oscillations we report in Figure 3. Perhaps we are again not understanding the reviewer correctly, as this does not seem an appropriate control for this experiment. In Figure 3 we show that CP110/Cep97 levels largely oscillate on the daughter centriole, not the mother centriole. So, the lack of a prominent oscillation on the mother centriole would already argue that the oscillation on the daughter centriole is not due to it moving up and down in the embryo during the nuclear cycle. Perhaps the reviewer is suggesting that the daughter centriole may move up and down, while the mother centriole does not? This seems extremely unlikely, as we know that mother and daughter centrioles remain tightly engaged throughout S-phase. Moreover, Asl cannot be used as a control to test this as Asl is only present on mother centrioles; thus, to show again that Asl levels do not oscillate on the mother centriole—as we showed previously (Novak et al., Curr. Biol., 2014)—is not a good control for the oscillation on the daughter centriole.

3. The reviewer asked why we feel it appropriate to quantify the ECL blots shown in Figure 8C, but not the blots shown in Figure 4A,B and Figure S1. We apologise for not explaining this properly. In the blots in Figure 8C we show that CP110 or Cep97 appear to be expressed at similar levels in different embryos under different conditions. When proteins are expressed at similar levels, the ECL blots show this quite well and consistently (e.g. the levels in each condition look similar no matter how long the exposure of the blot or how much secondary antibody is used). Thus, in this experiment, it is useful to perform this type of quantification to show that we have performed multiple repeats and the blots shown are representative of those repeats.

The blots in Figure 4A,B and Figure S1, however, show that various CP110- and Cep97- fusions are being expressed at *different* levels in the various lines we analyse here. In many control experiments over many years, we have found that quantifying such ECL blots is not an accurate way to estimate the *magnitude* of these differences (i.e. are the CP110/Cep97 proteins over- or under-expressed in the various lines by 2X, 5X or 10X compared to the endogenous proteins?). This is, at least in part, because, the absolute value of these differences can vary depending on how long the blot has been exposed, the amount of protein loaded and the concentration of secondary antibody used. Thus, we have found that the most reliable way to estimate the magnitude of these differences is to use serial dilutions of the extract containing the highest concentration of the relevant protein, and then estimate which dilution gives the most similar level of protein compared to the endogenous protein.

We have used this methodology routinely now for many years and in many publications without having to show the dilution blots. As the reviewer requests, we have now supplied examples of such serial dilution blots for each protein—RF#3 (Dilution Blots). In line with JCS policy, we have provided the original uncropped versions of all the other blots we use in the paper, but we have not currently included these dilution blots (as they are not formally a part of any of the Figures). It does not seem appropriate to show these serial- dilution blots in the actual data Figure, but we could include these in this Section of the Supplementary Material if the Reviewer thinks it crucial that we show them.

© 2022. Published by The Company of Biologists under the terms of the Creative Commons Attribution License (https://creativecommons.org/licenses/by/4.0/).

Dilution blots for CP110$^{O/E}$ and Cep97$^{O/E}$ constructs

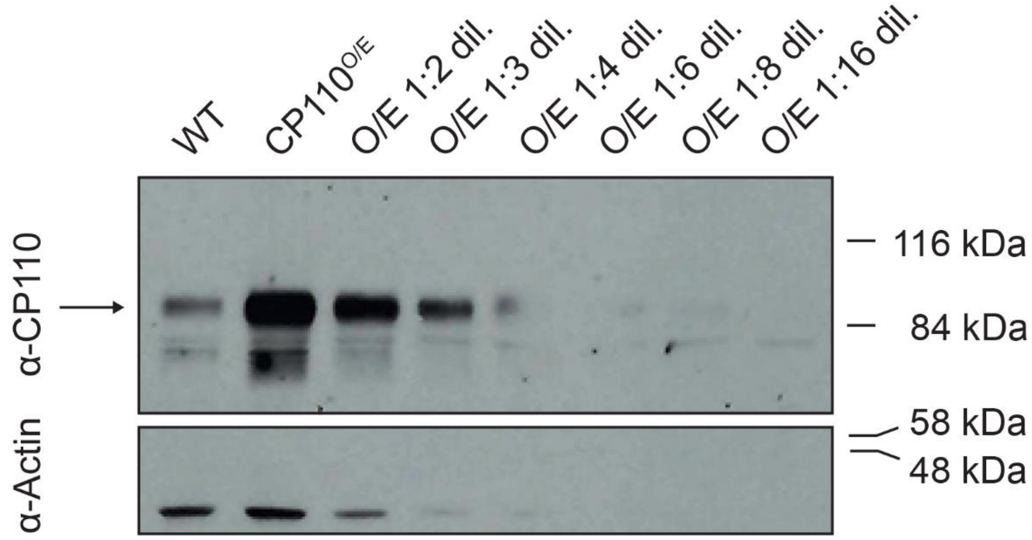

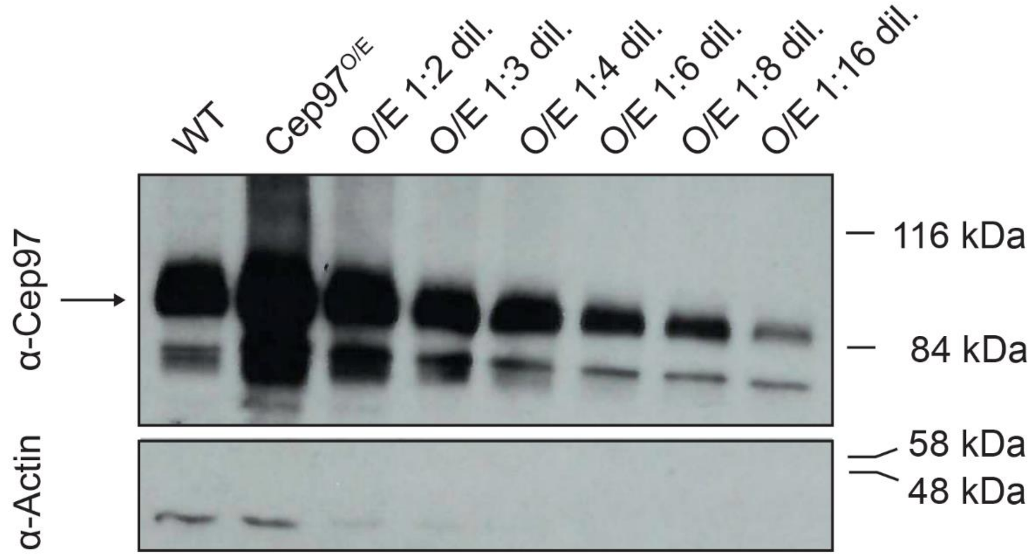

Other points:

The reviewer is correct that we should not have used the term “out of phase” to describe the difference in phase of the RFP-Cep97 and Plk4-GFP oscillations we show in Figure S2. We now simply state that the phases of the two oscillations are different, which is more accurate and is the most important point.

The reviewer suggests that the increase in the timing of the peak of the CP110 and Cep97 oscillations when we half the dose of Cyclin B are marginal, although significant (Figure 5E,F), and they suggest we adjust our description of this experiment accordingly (p11, para.2). While it is true that the time to peak measurement is only slightly, although significantly, extended in the Cyclin B half-dose embryos, it is very clear that the CP110 and Cep97 oscillations are extended in response to the extension in cell cycle length. Our description of this experiment is reasonably cautious, stating only that this result is consistent with our hypothesis that increasing Cdk/Cyclin

© 2022. Published by The Company of Biologists under the terms of the Creative Commons Attribution License (https://creativecommons.org/licenses/by/4.0/).

levels either directly or indirectly switch off the ability of the centrioles to recruit and/or maintain CP110/Cep97. Perhaps the Reviewer can suggest alternative wording that they feel more accurately reflects this data?

Reviewer #3

This Reviewer thought our revised manuscript was suitable for publication in JCS, although they made two comments.

First, they were confused as to how our radial measurements of the distribution of CP110/Cep97 around the centriole was relevant to the localisation of these proteins along the proximal-distal axis. We apologise for this confusion: the reviewer is correct, and we did not mean to imply this. We have now amended the Figure legend to clarify this point.

Second, the reviewer noted that using the fluorescence intensity of a structural proteins as a proxy for the physical length of the structure could be misleading, and they requested that we discuss this point. We agree, and now specifically mention that this assay has caveats and reference our original paper where our reasons for believing that Sas-6-GFP fluorescence is a reasonable proxy for centriole cartwheel length in these rapidly dividing embryos are discussed in more detail (p12, para.2). In addition, and in recognition of this point, we have toned down the strength of our conclusion that CP110/Cep97 influences cartwheel growth kinetics in several places throughout the manuscript.

We hope you and the Reviewers will agree that these changes have addressed any remaining concerns and that our manuscript is now suitable for publication at JCS.

Second decision letter

MS ID#: JOCES/2022/260015

MS TITLE: Centriole distal-end proteins CP110 and Cep97 influence cartwheel growth at the proximal-end of centrioles

AUTHORS: Mustafa G. G Aydogan, Laura E Hankins, Thomas L. L Steinacker, Mohammad Mofatteh, Saroj Saurya, alan wainman, Siu-Shing Wong, Xin Lu, Felix Y Zhou, and Jordan W Raff
ARTICLE TYPE: Research Article

Thank you for sending your manuscript to Journal of Cell Science through Review Commons.

I am happy to tell you that your manuscript has been accepted for publication in Journal of Cell Science, pending standard ethics checks. Thank you for your careful revisions and responses to the reviewers. I did not consider it necessary to return the manuscript to the reviewers.

© 2022. Published by The Company of Biologists under the terms of the Creative Commons Attribution License (https://creativecommons.org/licenses/by/4.0/).
